# Rapid interpretation of small-angle X-ray scattering data

**DOI:** 10.1371/journal.pcbi.1006900

**Published:** 2019-03-22

**Authors:** Marie Weiel, Ines Reinartz, Alexander Schug

**Affiliations:** 1 Department of Physics, Karlsruhe Institute of Technology, Karlsruhe, Germany; 2 Steinbuch Centre for Computing, Karlsruhe Institute of Technology, Eggenstein-Leopoldshafen, Germany; 3 Institute for Advanced Simulation, Jülich Supercomputing Center, Jülich, Germany; Virginia Tech, UNITED STATES

## Abstract

The fundamental aim of structural analyses in biophysics is to reveal a mutual relation between a molecule’s dynamic structure and its physiological function. Small-angle X-ray scattering (SAXS) is an experimental technique for structural characterization of macromolecules in solution and enables time-resolved analysis of conformational changes under physiological conditions. As such experiments measure spatially averaged low-resolution scattering intensities only, the sparse information obtained is not sufficient to uniquely reconstruct a three-dimensional atomistic model. Here, we integrate the information from SAXS into molecular dynamics simulations using computationally efficient native structure-based models. Dynamically fitting an initial structure towards a scattering intensity, such simulations produce atomistic models in agreement with the target data. In this way, SAXS data can be rapidly interpreted while retaining physico-chemical knowledge and sampling power of the underlying force field. We demonstrate our method’s performance using the example of three protein systems. Simulations are faster than full molecular dynamics approaches by more than two orders of magnitude and consistently achieve comparable accuracy. Computational demands are reduced sufficiently to run the simulations on commodity desktop computers instead of high-performance computing systems. These results underline that scattering-guided structure-based simulations provide a suitable framework for rapid early-stage refinement of structures towards SAXS data with particular focus on minimal computational resources and time.

## Author summary

Proteins are the molecular nanomachines in biological cells and thus vital to any known form of life. From the evolutionary perspective, viable protein structure emerges on the basis of a ‘form-follows-function’ principle. A protein’s designated function is inextricably linked to dynamic conformational changes, which can be observed by small-angle X-ray scattering. Intensities from SAXS contain low-resolution information on the protein’s shape at different steps of its functional cycle. We are interested in directly getting an atomistic model of this encoded structure. One powerful approach is to include the experimental data into computational simulations of the protein’s function-related physical motions. We combine scattering intensities with coarse-grained native structure-based models. These models are computationally highly efficient yet describe the system’s dynamics realistically. Here, we present our method for rapid interpretation of scattering intensities from SAXS to derive structural models, using minimal computational resources and time.

## Introduction

Protein structure determination is a key challenge in modern biophysics and ultimately aims at revealing a fundamental relation between biomolecular structure and function. To date, the Protein Data Bank [1] comprises an ever-expanding variety of almost 150 000 macromolecular structures from experimental methods like X-ray diffraction analysis and nuclear magnetic resonance (NMR) spectroscopy. Upon combination with biomolecular simulation, static structural models can be extended by dynamic snapshots even for complex nanomachines such as the ribosome [2, 3]. Concurrent progress in experiment and simulation has led to coupling of complementary techniques within hybrid methods, emerging as a new paradigm to accelerate such procedures and further improve quality of their results [4–8]. Small-angle X-ray scattering (SAXS) is an experimental technique for structural characterization of biomolecules in solution and complementary to high-resolution methods such as X-ray crystallography and NMR spectroscopy. X-ray scattering studies can be conducted under various conditions and provide information on both steady-state structure and kinetics of molecular reactions down to a spatial resolution of 50 to 10 Å [9]. Using synchrotron-based wide-angle X-ray scattering, transiently populated conformations can be measured midst-movement on a timescale of 100 ps without size limitations inherent in NMR and electron microscopy studies [9]. X-ray free electron lasers even allow for ultrashort and extremely brilliant X-ray pulses of a few tens of femtoseconds [10]. In biological SAXS, a dilute solution of macromolecules is exposed to a monochromatic X-ray beam. A reaction is triggered, e.g. by ligand binding, and the elastically scattered intensity is recorded in the small-angle regime at subsequent points in time. For spatially isotropic particle distributions, these intensity profiles directly yield low-resolution information on averaged molecular size and shape. Experimental data is typically processed in form of difference curves, where the initial intensity serves as a reference and is subtracted from that of a certain time point. Such curves reflect a difference in pair distribution functions and thus structural change during the molecular reaction. In a SAXS profile, the number of independent data points generally equals the number of independent Shannon channels [9, 11]. With spherically averaged scattering intensities containing only tens of such points, their information content is insufficient to determine all degrees of freedom and infer a three-dimensional molecular model without prior physical knowledge. To date, SAXS data are often interpreted by ab initio reconstruction of low-resolution envelopes from one-dimensional scattering intensities [12–14]. Due to a fundamental ambiguity in the inverse problem of SAXS, uniqueness of the resulting models cannot be ensured. In particular with large structural rearrangements being involved, such methods do not yield reliable results. Other approaches such as rigid body refinement [15, 16], simulated annealing of dummy atom collections [13, 17], and targeted selection of suitable frames from biomolecular simulations [10, 18, 19] rely on sequential sampling and comparison with experimental data by generating candidate structures and calculating their respective scattering patterns. These methods have a non-negligible risk of failing to find the correct structure owing to the inherent ambiguity and limited sampling of conformational space.

A powerful approach for interpretation of low-information experimental data is to integrate it into a computational description of the molecule’s physical movements. Here, X-ray scattering intensities are included into biomolecular simulations via a differentiable pseudo-energetic bias term favoring those configurations consistent with the data. A given starting structure is refined towards SAXS curves, while underlying force fields provide the required physico-chemical pre-knowledge. Thermal ensembles of proteins in solution can be sampled, whereas simultaneously having regard to the structural information from SAXS. This yields a selection of physically reasonable conformations in accordance with the input scattering curve. With the aid of supplementary experimental information, structural transitions are accelerated and potential force field biases reduced. Approaches to include SAXS data into explicit-solvent molecular dynamics (MD) already exist and recent studies highlight the great potential of combining experimental scattering data with computational simulations to interpret biomolecule dynamics in solution [5, 20, 21]. However, accurate description of large-scale conformational motions remains a technical challenge in (biased) MD as a result of prohibitive computational costs.

To overcome these issues, numerous efforts to reduce the system’s effective degrees of freedom have been made by coarse-graining either the structural resolution or the force field applied [22]. In this work, our focus is set on native structure-based models (SBMs) [23–26], which probe dynamics arising from the system’s native geometry. According to energy landscape theory [23, 27] and the principle of minimal frustration [28], proteins feature an evolutionarily smoothened free energy funnel. SBMs define the native state to be in the funnel’s global energetic minimum. As a result, an overall energetic drive to the native structure overtops kinetic traps originating from non-native interactions. Giving access to biologically relevant time and length scales, SBMs provide rich information on complex processes, including major structural changes. Sampling can be improved by drastically decreasing force field complexity without loss of principal information on the system’s characteristics. Thus it is even possible to perform instructive atomistic simulations [29, 30] on desktop PCs. Successful applications cover a wide range of protein dynamics, including folding pathways [31–34] and kinetics [35]. SBMs are also employed for structure prediction [36–38], integrative structural modeling of experimental data from e.g. FRET [4] and cryo-EM [6], and investigation of transition state ensembles [39, 40]. Conformational transitions involving multiple stable states can be described using so-called multi-Gō models [41].

We systematically research how experimental scattering data can be incorporated into robust theoretical models, which at the same time support full molecular flexibility and accurately describe the dynamics of complex biomolecules. SBM simulations provide an easily extendible framework for this and allow to efficiently integrate and interpret intensity profiles from biological solution scattering experiments. We validate our method by investigating structural transitions induced by artificial scattering data in three two-state protein systems. Achieving equal-quality results, we find that computational efforts reduce by more than two orders of magnitude compared to explicit-solvent MD-based methods.

### Theory

#### Small-angle X-ray scattering

In SAXS, a solution of macromolecules is exposed to X-rays with wavelength λ. The integrated scattered intensity *I* is recorded in the small-angle regime as a function of momentum transfer *q* = 4*π* sin *θ*/λ, where 2*θ* is the angle between incident and scattered radiation. Random positions and orientations of solute molecules result in an isotropic intensity distribution, which, for monodisperse non-interacting particles, is proportional to the spatially averaged scattering from a single particle [42, 43]. The net particle scattering, in turn, is related to the contrast determined by the electron density difference between solute and solvent.

Mathematically, the spatially averaged scattering from a molecule described as a discrete sum of elementary scatterers can be modeled by the Debye equation [44]:

$$\begin{array}{c} I\left(q\right)=\sum_{i,j}^{N}f_{i}\left(q\right)f_{j}\left(q\right)\frac{\text{sin}(qr_{ij})}{qr_{ij}} \end{array}$$ (1)

Here, *f*_*i*_ is the form factor of scatterer *i* and *r*_*ij*_ the distance between scatterers *i* and *j*.

As the Debye summation is an $O(N^{2})$ problem, a fast method for repeated evaluation of SAXS profiles from atomistic structural models is a top priority for dynamic structural refinement procedures. That is why scattering calculations in our work use a coarse-grained protein representation with effective residue-based form factors corrected for displaced solvent [45–47]. In this form, Eq 1 does not account for the fact that the solvent density in the macromolecular hydration shell generally differs from its bulk value. However, the hydration shell scattering is typically several orders of magnitude smaller than the solute and excluded-solvent scattering [48]. In addition, systematic errors and solvation layer contributions effectively cancel out upon taking intensity differences. Thus, less sophisticated solvation treatment can be used to reliably model difference data [20, 46]. A more detailed introduction to molecular solution X-ray scattering theory can be found in S1 Appendix.

#### Native structure-based models

Structure-based models (SBMs), also referred to as Gō-type models, provide a minimalistic description of biomolecular dynamics arising from funneled energy landscapes. To a first approximation, long-range interactions between remote residues are governed by the protein’s geometry [24, 26]. Stable native structure is principally associated with minimal free energy. Energy landscape theory [23, 27] and the principle of minimal frustration [28] explain robust structure formation to be induced by minimally frustrated native interactions, giving rise to the typical energy funnel [23]. Resulting dynamics are modeled based on the assumption that native interactions are generally stabilizing, whereas non-native interactions are only incorporated to preserve proper excluded volume. The SBM’s essential part is founded in its contact potential. Native contacts are defined by pair interactions between spatially proximate atoms of residues *i* and *j* in the native structure, where *i* > *j* + 3. To stabilize the initial structure’s native fold, each contact is assigned a Lennard-Jones-like potential comprising an attractive as well as a repulsive term. Other non-local interactions are purely repulsive [24]. Thus, the all-atom structure-based potential explicitly represents the biomolecule’s atomic geometry. With native bond lengths *r*_0_, bond angles *ϑ*_0_, and proper and improper dihedral angles *ϕ*_0_ and *χ*_0_, it reads [29]:

$$\begin{array}{c} \begin{array}{cc} V_{\text{SB}}= & \sum_{\text{bonds}}K_{b}{\left(r-r_{0}\right)}^{2}+\sum_{\text{angles}}K_{a}{\left(\vartheta -\vartheta_{0}\right)}^{2}+\sum_{\text{improper}\;\text{dihedrals}}K_{i}{\left(\chi -\chi_{0}\right)}^{2}\\ + & \sum_{\text{dihedrals}}K_{d}[(1-\text{cos}(\phi -\phi_{0}))+\frac{1}{2}(1-\text{cos}(3(\phi -\phi_{0})))]\\ + & \sum_{\text{contacts}}K_{c}[{\left(\frac{\sigma_{ij}^{0}}{r_{ij}}\right)}^{12}-2\cdot {\left(\frac{\sigma_{ij}^{0}}{r_{ij}}\right)}^{6}]\\ + & \sum_{\text{non-native}\;\text{contacts}}K_{\text{nc}}{\left(\frac{\tilde{\sigma }}{r_{ij}}\right)}^{12} \end{array} \end{array}$$ (2)

Energetic weights of bonds, angles, improper dihedral angles, and non-native contacts are *K*_b_ = 20 000 *ε*/nm^2^, *K*_a_ = 40 *ε*/deg, *K*_i_ = 40 *ε*/deg, and *K*_nc_ = 0.01 *ε*, respectively. *ε* gives the SBM’s reduced energy unit and deg refers to arc degree. Weights for contact potential *K*_c_ and proper dihedral angle potential *K*_d_ are derived as in Ref [49]. $\sigma_{ij}^{0}$ is the native distance of atom pair (*i*, *j*) in contact, *r*_*ij*_ the actual distance between atoms *i* and *j*, and $\tilde{\sigma }$ the excluded volume for Pauli repulsion. The dihedral potential allows for occupation of isomeric conformations next to the native state. We use atomistic SBMs, which explicitly include all non-hydrogen atoms as unit beads with equal masses, radii, and force constants. Solvent and hydrogen atoms are only treated implicitly [29, 50].

SBMs employ reduced units, i.e. length scale, time scale, mass scale, and energy scale are all 1. GROMACS naturally uses a nm length scale, ps time scale, amu mass scale, and kJ/mol energy scale. While the PDB Å length scale can easily be converted into nm, mass scale, time scale, and energy scale remain free. In principle, it is possible to determine a system-specific overall energy and mass scale from the structure and its dynamics and subsequently infer a time scale. As energetic roughness possibly decelerating a real system’s dynamics are not considered, this should be performed with great care. Alternatively, time scales can be extracted by comparing simulation results with experimental observations using e.g. folding rates or rotational correlation times [4]. There is no standard method of calculating ‘real’ times from structure-based simulations established. Since absolute time plays a subordinate role for the aim of this work, simulation time is specified in arbitrary units (arb). As a result of the system’s inherently accelerated dynamics, the ‘real’ time unit in SBMs is certainly longer than the ps timescale reported by GROMACS [49]. Temperature sets an energy scale *ε* = *k*_B_*T* and is reported in reduced GROMACS units throughout. For a detailed discussion on SBM units, see Ref [49].

#### Bias potential and force calculation

To bias a simulation towards conformations reproducing a certain scattering curve, the SBM potential *V*_SB_ is extended by the term *V*_XS_ depending on the Debye equation [20]:

$$\begin{array}{c} \begin{array}{cc} V & =V_{\text{SB}}+V_{\text{XS}}=V_{\text{SB}}+\frac{k_{\chi }}{2}\chi^{2}\\ \text{with}\;\chi^{2} & =\sum_{q}{\left[\frac{\Delta I_{\text{exp}}\left(q\right)-\alpha \left\{I_{\text{calc}}\left(q\right)-I_{\text{ref}}\left(q\right)\right\}}{\sigma_{q}}\right]}^{2} \end{array} \end{array}$$ (3)

*I*_calc_(*q*) and *I*_ref_(*q*) are the theoretical scattering intensity of the simulation’s current conformation and the reference intensity obtained from the initial structure, respectively. Δ*I*_exp_(*q*) is a difference scattering curve either measured experimentally or calculated via theoretical absolute scattering curves. *k*_*χ*_, which is specified in the SBM’s reduced energy unit *ε*, gives the relative weighting factor of the scattering bias *V*_XS_ with respect to the structure-based potential *V*_SB_. The weights *σ*_*q*_ of individual *q* points in the scattering curve are calculated from experimental errors. Herein, errors in the difference data *σ*_Δ_(*q*) are preferred over errors in the reference curve *σ*_ref_(*q*), i.e.

$$\begin{array}{c} \sigma_{q}=\frac{\sigma_{\Delta }\left(q\right)}{\Delta I_{\text{exp}}\left(q\right)}+1\;\text{or}\;\sigma_{q}=\frac{\sigma_{\text{ref}}\left(q\right)}{I_{\text{ref}}\left(q\right)}+1, \end{array}$$ (4)

respectively. Given no experimental errors, all *σ*_*q*_ are set to 1. As the data are more and more affected by experimental errors with increasing scattering angle, wide-angle scattering data are naturally given less weight compared to small-angle scattering data by this means. *α* is the fraction of the observed sample undergoing conformational change given by the relative yield of the difference experiment. Thus, *χ*^2^ can be considered a dissimilarity measure of the current conformation’s scattering in the simulation with respect to the target data.

The potential in Eq 3 was recently implemented in the popular MD package GROMACS 5 [20]. It provides a strong basis for interpretation of SAXS data within SBM simulations. The Debye summation is an $O(N^{2})$ problem and thus becomes computationally expensive for large biomolecules. To match the level of structural detail with the intrinsic low-resolution and coarse-grained nature of solution scattering data, we combined SBMs with residue-based calculations of Debye scattering curves. Intensity profiles of current conformations were computed on-the-fly using amino-acid scattering factors corrected for displaced solvent [20, 47]. Computational costs thus could be reduced to a minimum.

### Studied systems

We investigated structural transitions in three two-state protein systems, where the target structure was initially known.

#### Villin headpiece

**Fig 1:**
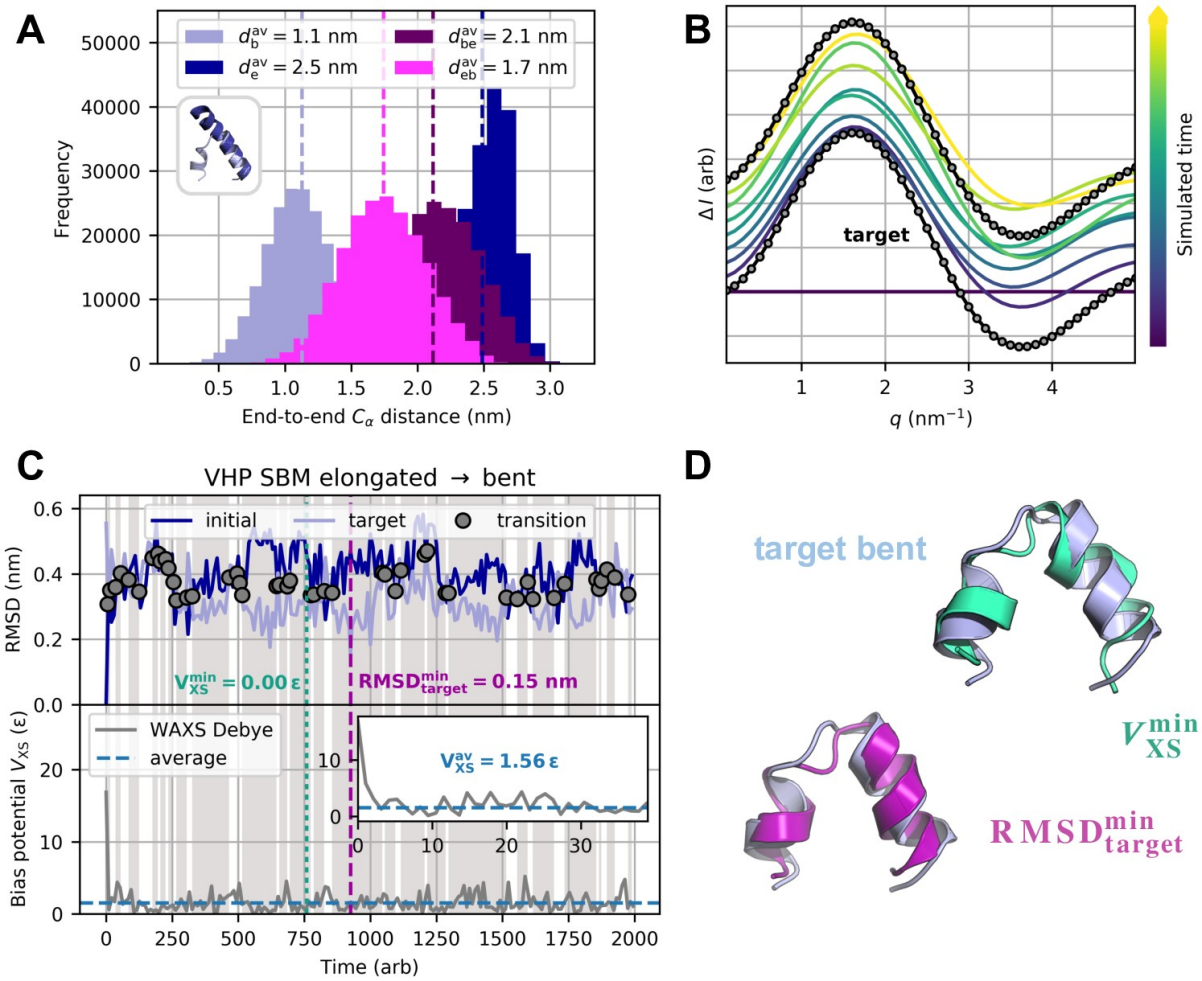
Proof-of-concept simulations for VHP-based polypeptide system. Results are shown for parameters (*T*, *k*_*χ*_) = (90, 5 ⋅ 10^−8^ *ε*). (A) End-to-end C_α_ distance distributions. Inset: Bent (light blue) and elongated (dark blue) conformation have inter-terminal distances of 1.07 nm and 2.68 nm, respectively. $d_{b}^{\text{av}}$, $d_{e}^{\text{av}}$, $d_{\text{eb}}^{\text{av}}$, and $d_{\text{be}}^{\text{av}}$ denote average end-to-end distances for free bent (light blue), free elongated (dark blue), scattering-guided elongated-to-bent (magenta), and scattering-guided bent-to-elongated (purple) SBM simulations. (B) Artificial difference scattering data for elongated-to-bent transition. 50 equidistant *q* points between 0.1 nm^−1^ and 5 nm^−1^ were included (grey circles). Debye intensities of structures from equidistant time points in the simulation (blue to yellow) are displayed with small offsets. (C) Initial and target RMSD (top) and bias energy (bottom) versus simulated time. With both initial (elongated, dark blue) and target (bent, light blue) RMSD proceeding in close proximity, multiple structural transitions (grey circles) occurred back and forth. *V*_XS_ initially decreased significantly and further exhibited small-scale fluctuations throughout the refinement. (D) Best structures in terms of minimum target RMSD and bias energy. ${\text{RMSD}}_{\text{target}}^{\text{min}}$ (purple) and $V_{\text{XS}}^{\text{min}}$ (turquoise) structure have target RMSDs of 0.15 nm and 0.27 nm, respectively.

A basic approach to elucidating the mechanisms of protein folding and conformational transitions is to study short sequences with fast folding kinetics. The actin-binding protein villin consists of multiple domains capped by a C-terminal headpiece. As a proof of principle, we set up a small test system by extracting the 21-amino-acid subregion between residues 54 and 74 from the NMR structure of villin headpiece (VHP, PDB code 1VII [51]). This polypeptide subdomain consists of two short helices connected by a β-turn and is referred to as the bent conformation. The elongated conformation was constructed as a perfect α-helix with identical sequence in PyMOL (Fig 1A inset) [52]. The root-mean-square deviation (RMSD) on C_α_ level between bent and elongated state is 0.55 nm. We used this two-state system to probe how the conformational distribution of a polypeptide can be influenced by biasing biomolecular simulations towards configurations reproducing a theoretical scattering curve. Note that this test case constitutes a very difficult one in geometry-derived SBMs due to the radical change in secondary structure.

#### Adenylate kinase

**Fig 2:**
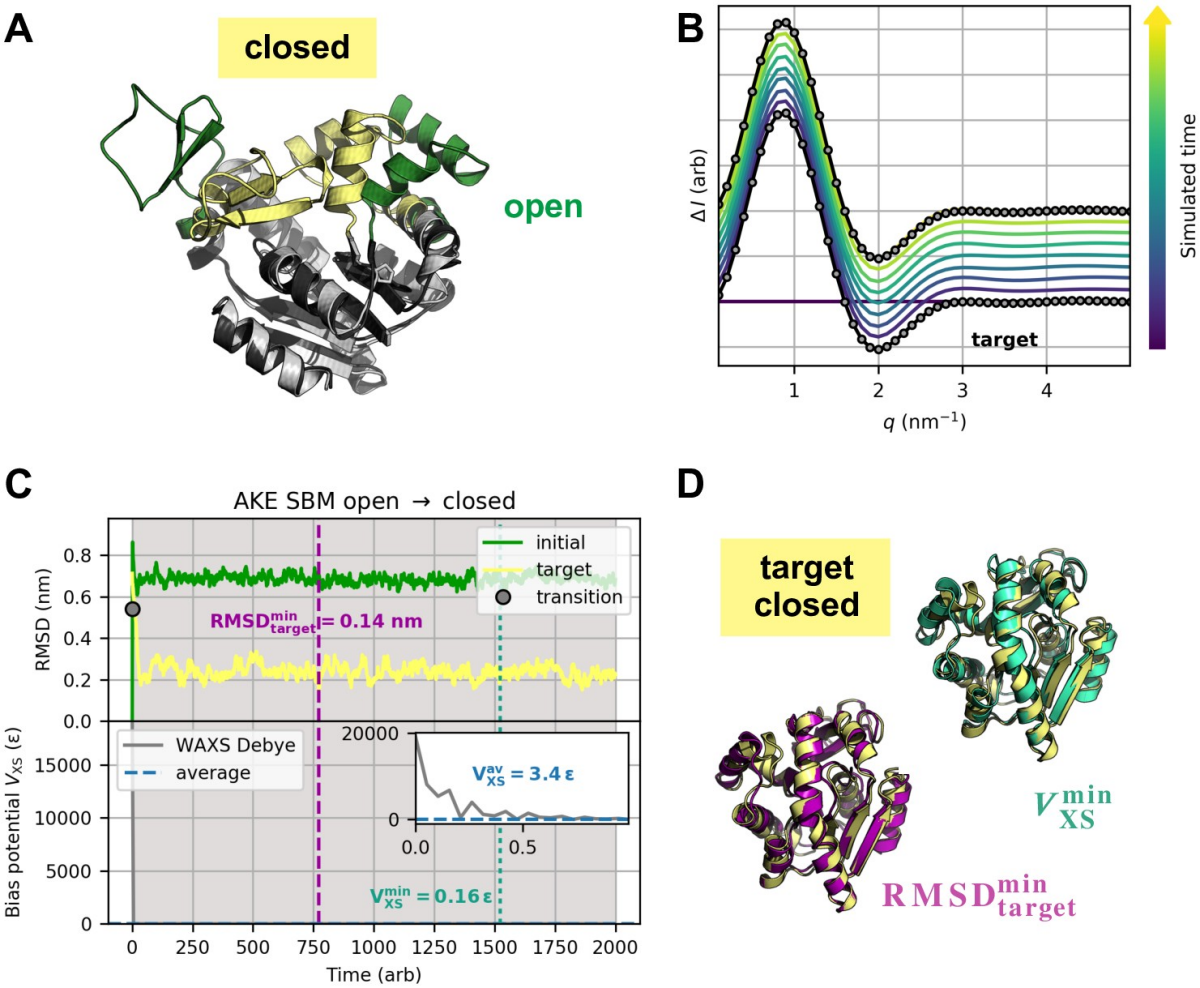
Method validation using the example of AKE’s open-to-closed transition. Results are shown for parameters (*T*, *k*_*χ*_) = (50, 8 ⋅ 10^−9^ *ε*). (A) AKE test system. Open (green) and closed (yellow) conformations with CORE domain (grey) spatially aligned. (B) Artificial target data (grey circles) were computed from absolute Debye intensities. Theoretical difference curves of simulated structures (blue to yellow) almost perfectly match the target data and were plotted with small offsets. (C) Initial and target RMSD (top) and bias energy (bottom) versus simulated time. (D) Best structures as measured by target RMSD and bias energy. ${\text{RMSD}}_{\text{target}}^{\text{min}}$ structure (purple) and $V_{\text{XS}}^{\text{min}}$ structure (turquoise) exhibit target RMSDs of 0.14 nm and 0.20 nm, respectively.

Adenylate kinase (AKE) is a signal-transducing phosphotransferase enzyme catalyzing the interconversion of adenine nucleotides ATP + AMP ⇌ 2 ADP. This three-domain protein, comprising a large central CORE domain flanked by a LID and an NMP domain, has two distinct binding sites and plays a key role in cellular energy homoeostasis. The reversible transition between AKE’s open (PDB code 4AKE [53]) and closed (PDB code 1AKE [54]) conformation (Fig 2A) is quintessential to its catalytic function [55] and directly related to competing native interactions of the respective states. The C_α_ RMSD between open and closed structure is 0.71 nm. Scattering-guided simulations started from open and closed conformation and aimed at closed and open state, respectively.

#### Lysine-arginine-ornithine binding protein

**Fig 3:**
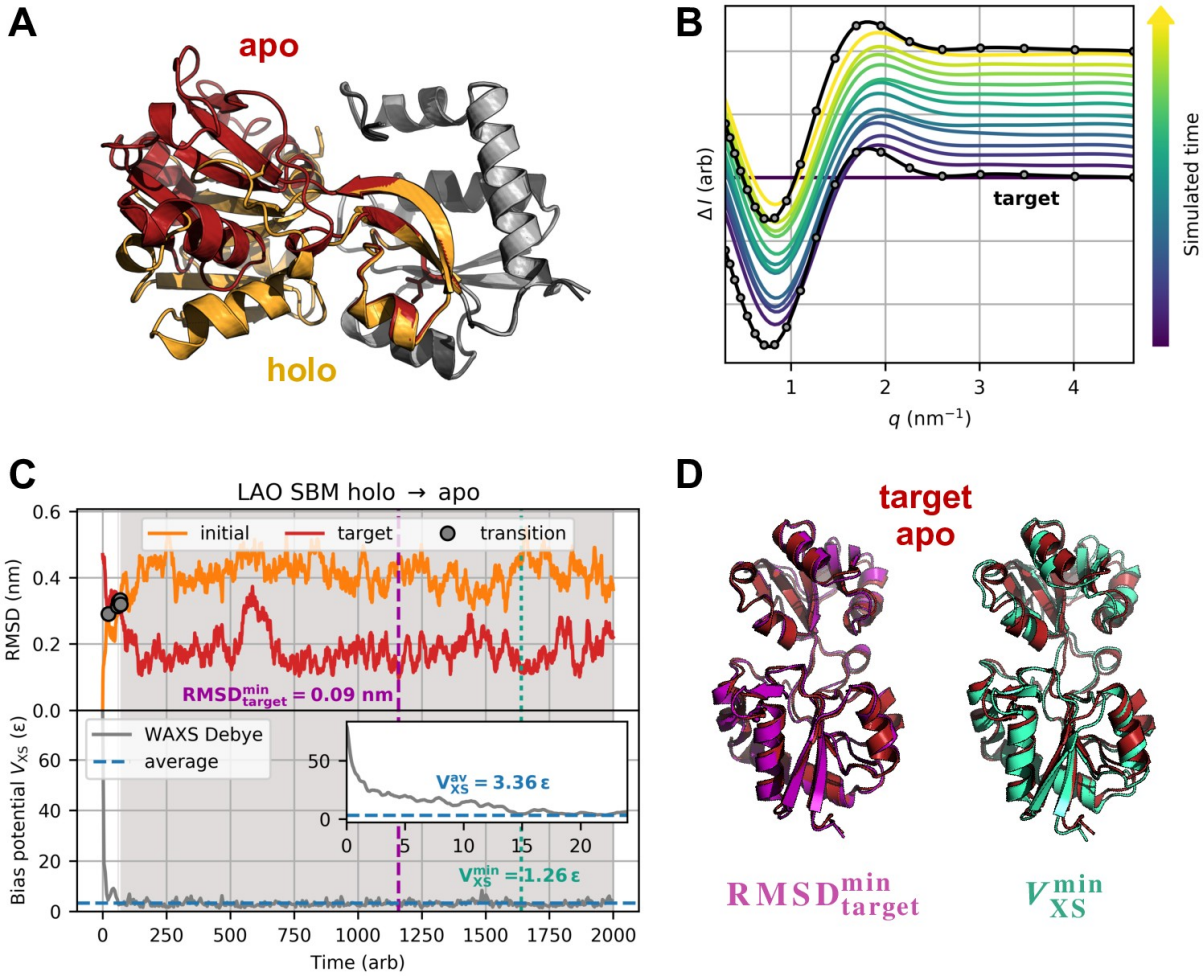
Method validation using the example of LAO protein’s holo-to-apo transition. Results are shown for parameters (*T*, *k*_*χ*_) = (50, 9 ⋅ 10^−11^ *ε*). (A) LAO protein test system. LAO protein undergoes a large-scale conformational transition upon ligand binding, meaning the lobes are clearly separated in the unliganded state (apo form, red), whereas in mutual contact with lysine ligand bound (holo form, orange). Congruent parts in holo and apo conformation are depicted in grey. (B) Artificial target data for the holo-to-apo transition (grey circles). 20 *q* points between 0.3 nm^−1^ and 4.6 nm^−1^ were included. Theoretical difference intensities of simulated structures (blue to yellow) were plotted with small offsets and approached the target curve in the course of the simulation. (C) Initial and target RMSD (top) and bias energy (bottom) versus simulated time. The refinement involved one instantaneous transition from initial holo to target apo state in terms of intersecting RMSD curves (grey dots). With the simulation approaching its designated target state, the bias potential minimized accordingly. (D) Best structures as measured by target RMSD and bias potential. ${\text{RMSD}}_{\text{target}}^{\text{min}}$ structure (purple) and $V_{\text{XS}}^{\text{min}}$ structure (turquoise) exhibit target RMSDs of 0.09 nm and 0.16 nm, respectively.

Lysine-arginine-ornithine binding (LAO) protein undergoes large-scale conformational change upon ligand binding. Both apo (PDB code 2LAO [56]) and holo form (PDB code 1LST [56]) consist of two lobes connected by two short strands (Fig 3A). Whereas these lobes are clearly separated in the unliganded state, they are in contact when lysine-liganded. During the conformational transition, one domain effectively rotates around a hinge axis defined by two points on adjacent β-strand termini [56]. Unliganded holo and apo structure exhibit a C_α_ RMSD of 0.47 nm. Scattering-guided simulations started from the holo state and aimed at the apo state, and reversed.

## Results and discussion

We tested our method for SBM refinement of protein structures towards scattering data for three two-state systems. Artificial difference data were derived from absolute intensities by subtracting the initial reference scattering from the target curve. Root-mean-square deviation (RMSD) was employed as a quantitative measure for distinction of structurally different configurations. We calculated the trajectory’s time-dependent RMSD with respect to initial and target structure on C_α_ level, referred to as initial and target RMSD. Computation time $\tau_{0.2}^{\text{comp}}$ related to the trajectory approaching a structure with target RMSD less than 0.2 nm was extracted as an efficiency estimate. The bias energy *V*_XS_ was analyzed as a function of simulated time. To assess its suitability as a reliable identifier of physically reasonable structures matching the target data, we examined its Pearson correlation *ρ* with the target RMSD. Results are shown for parameter combinations of temperature and coupling strength optimized via grid-search variational studies. Minimum target RMSD, average bias potential, and average *χ*^2^ (see Eq 3) were extracted as functions of bias weight in the range of 10^−11^ *ε* and 10^−7^ *ε* at temperatures 50, 70, 90, and 110. We considered minimum target RMSD as a primary indicator to assess whether a simulation converges sufficiently close towards the target to provide a real chance to observe the conformations of interest despite the SBM’s strong bias towards the native state. Together with the bias potential, ${\text{RMSD}}_{\text{target}}^{\text{min}}$ can be used to estimate the extent of structural convergence within one simulation. Additionally to *V*_XS_, we analyzed the average *χ*^2^ dissimilarity of scattering curves from a simulation with respect to the target data. This parameter was used to evaluate the extent of convergence on the pure data level among simulations using different parameter combinations.

Furthermore, we conducted analogous scattering-guided explicit-solvent MD simulations for a comparative performance check. To ensure structural conformity between ensembles generated by SBM and explicit-solvent MD, we calculated both radius of gyration and asphericity as functions of simulated time (see S2 Appendix and S1, S2 and S3 Figs). A detailed description of simulation set-ups is provided in Materials and methods.

### Villin headpiece

For the VHP-based polypeptide system (Fig 1A inset), proof-of-concept simulations aimed at both transitions from bent to elongated and elongated to bent conformation. Target scattering data were calculated via the Debye equation (Eq 1). We analyzed the backbone’s elongatedness by extracting distances between N-terminal and C-terminal C_α_ atoms for both free and scattering-guided SBM simulations. The distance distributions (Fig 1A) show a clear shift towards the target structure’s end-to-end distance. This confirms that, as intended, conformations which are not in accordance with the target curve are avoided in scattering-guided simulations. To show the degree of similarity between typical X-ray scattering patterns from the refinement and the target data, Debye intensities of simulated structures are illustrated in Fig 1B. The curves converge to a certain extent, but do not show perfect agreement. This is due to the fact that the refinement is not only steered by the scattering bias, but also by the physico-geometrical SBM, so that an equilibrium between these two contributions settles in. Computation time scaled as 1.4 to 1 for scattering-guided and free SBM simulations. As the Debye summation is an $O(N^{2})$ problem (see Eq 1), the ratio of scattering-guided and free computation times will substantially increase with system size. In this context, rapid evaluation of SAXS profiles from structural models becomes even more important for dynamic refinement procedures such as scattering-guided biomolecular simulations.

**Fig 4:**
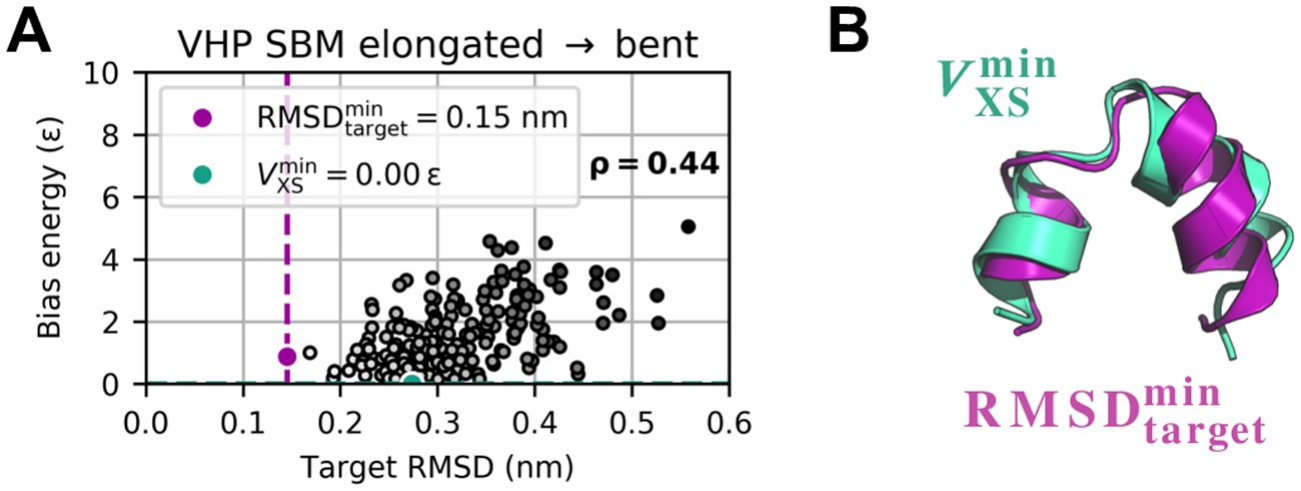
Bias potential versus target RMSD for elongated-to-bent transition. (A) Bias potential versus target RMSD. (B) ${\text{RMSD}}_{\text{target}}^{\text{min}}$ (purple) and $V_{\text{XS}}^{\text{min}}$ (turquoise) structure have a C_α_ RMSD of 0.25 nm with respect to each other.

Time-dependent RMSD curves and bias potential are depicted in Fig 1C and Panel A of S4 Fig for elongated-to-bent and bent-to-elongated transition, respectively. Guiding the simulations towards the target scattering data obviously causes the structural transition to bidirectionally occur back and forth. Minimum target RMSDs are 0.15 nm (Fig 1D) and 0.19 nm (Panel B in S4 Fig), respectively. This implies that structure-opening conformational changes from rather compact to more spacious structures are more difficult to sample than structure-closing ones. Free SBM simulations of bent and elongated conformation yielded average C_α_ RMSDs of 0.17 nm and 0.18 nm, respectively. In light of this, scattering-guided SBM simulations were capable of reproducing each target structure with the method’s inherent best possible accuracy. Despite the drastic change in secondary structure, they could model the conformational transition in both directions properly and persistently sample physically reasonable structures near the target conformation. Result parameters are summarized in Table 1 along with the values from analogous explicit-solvent MD simulations. Considering computation times $\tau_{0.2}^{\text{comp}}$, the structure-based method turned out to be faster by two orders of magnitude than the full-MD approach in terms of wall-clock time. Detailed explicit-solvent MD results can be found in S5 and S6 Figs. The bias energy was analyzed as a function of the trajectory’s target RMSD. As displayed in Fig 4A, low bias potential is principally associated with low target RMSD. A Pearson correlation *ρ* of 0.44 indicates that they are in fact positively correlated. However, we find a considerably spread ensemble of distinct structures at equal bias potential levels. With a bias potential of 0.88 *ε*, the minimum target RMSD structure is not exactly in the energetic minimum. Results for the reverse transition are presented in Panels C and D in S4 Fig. Analogous explicit-solvent MD results can be found in Panels C and D in S5 and S6 Figs.

**Table 1:** Result parameters for scattering-guided simulations of VHP.

method	SBM		MD	
transition	e → b	b → e	e → b	b → e
*k*_*χ*_	5 ⋅ 10^−8^ *ε*		5 ⋅ 10^−9^ kJ/mol	
*T*	90		330 K	
${\text{RMSD}}_{\text{target}}^{\text{min}}\;\left(\text{nm}\right)$	0.15	0.19	0.08	0.25
RMSD_initial_(nm)	0.47	0.54	0.55	0.47
${\text{RMSD}}_{\text{free}}^{\text{av}}\;\left(\text{nm}\right)$	0.17	0.18	0.29	0.26
$\tau_{0.2}^{\text{comp}}\;\left(\text{hh}:\text{mm}:\text{ss}\right)$	00: 00: 57	00: 01: 05	02: 58: 05	-
$V_{\text{XS}}^{\text{av}}$	1.56 *ε*	1.00 *ε*	3.78 kJ/mol	0.79 kJ/mol
$V_{\text{XS}}^{\text{min}}$	0.00 *ε*	0.00 *ε*	1.82 kJ/mol	0.39 kJ/mol
${\text{RMSD}}_{\text{target}}\left(V_{\text{XS}}^{\text{min}}\right)\;\left(\text{nm}\right)$	0.27	0.37	0.24	0.39
$\text{RMSD}\left({\text{RMSD}}_{\text{target}}^{\text{min}}\;\text{-}\;V_{\text{XS}}^{\text{min}}\right)\;\left(\text{nm}\right)$	0.27	0.31	0.21	0.28

Elongated-to-bent and bent-to-elongated transition are referred to as e → b and b → e, respectively. Minimum target RMSD (${\text{RMSD}}_{\text{target}}^{\text{min}}$), associated initial RMSD (RMSD_initial_), average RMSD from a corresponding free simulation of each target (${\text{RMSD}}_{\text{free}}^{\text{av}}$), computation time associated with a target RMSD less than 0.2 nm ($\tau_{0.2}^{\text{comp}}$), average bias potential ($V_{\text{XS}}^{\text{av}}$), minimum bias potential ($V_{\text{XS}}^{\text{min}}$), target RMSD associated with minimum bias energy (${\text{RMSD}}_{\text{target}}\left(V_{\text{XS}}^{\text{min}}\right)$), and the RMSD between minimum target RMSD structure and minimum bias energy structure ($\text{RMSD}({\text{RMSD}}_{\text{target}}^{\text{min}}\;\text{-}\;V_{\text{XS}}^{\text{min}})$) are listed.

**Fig 5:**
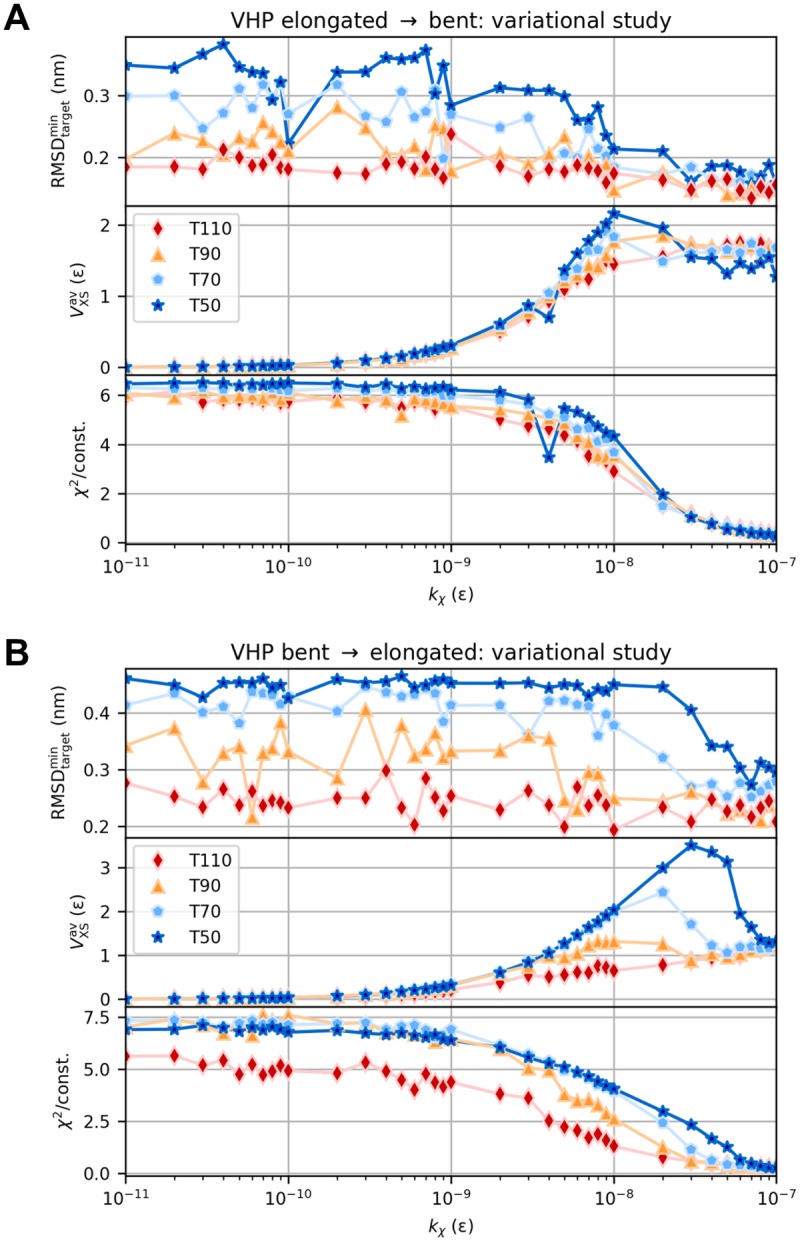
Variational study for VHP-based test system. Minimum target RMSD, average bias energy, and average *χ*^2^ dissimilarity for elongated-to-bent (A) and bent-to-elongated (B) transition as a function of coupling strength *k*_*χ*_ at different temperatures *T*. The variational series comprised 296 simulations in total.

To examine the influence of temperature and bias weight, we conducted grid-search variational studies. Results are depicted in Fig 5A and 5B for elongated-to-bent and bent-to-elongated transition, respectively. As soon as the initially increasing $V_{\text{XS}}^{\text{av}}\left(k_{\chi }\right)$ drops down (*T* = 50, 70) or plateaus (*T* = 90, 110), ${\text{RMSD}}_{\text{target}}^{\text{min}}$ converges towards the average value of related free simulations (see Table 1). Average *χ*^2^ dissimilarity of simulated scattering curves with respect to the target data minimizes accordingly. Near these turnaround points labeled $k_{\chi }^{*}$ hereafter, structure-based potential and scattering bias are assumed to be thoroughly balanced. This promotes rapid conformational transitions according to the target data in due consideration of the physico-geometrical model, but prevents the data from being overfitted. In SBMs, the bias potential has to be weighted in such a manner as to introduce a distinct competing minimum to the original single-basin energy funnel. We set $k_{\chi }∼O\left(k_{\chi }^{*}\right)$ to ensure occurrence of a clear transition, whilst modifying the underlying regular potential as little as possible. The elongated-to-bent transition yielded a smaller $k_{\chi }^{*}\approx 1\cdot 10^{-8}\;\varepsilon$ (see Fig 5A) compared to the bent-to-elongated case with $k_{\chi }^{*}\approx 5\cdot 10^{-8}\;\varepsilon$ (see Fig 5B). This behavior confirms our previous finding of structure-closing transitions to be favored over structure-opening ones.

For both transitions, we find the effect of gradually increasing the coupling constant to be less pronounced at higher temperatures (Fig 5). It is conspicuous that, independent of *k*_*χ*_, all simulations at *T* = 110 could—at least temporarily—sample conformations near the target. The increased thermal energy allows to overcome potential barriers in the energy landscape, resulting in greater protein flexibility and sampling power. However, these thermal structural fluctuations by itself are isotropic in conformational space and not directed towards any particular conformation as is the case with a major scattering bias. In contrast, at lower temperatures *T* = 50 and 70, ${\text{RMSD}}_{\text{target}}^{\text{av}}$ values almost double with *k*_*χ*_ increasing up to the order of 10^−8^ *ε*, before they significantly decline as well. With less thermal energy being available and an increased coupling of simulations to scattering intensities attaching more relative importance to structural information from SAXS, this is in accordance with the expectations. Remember that the global change in orientation of secondary structure elements with respect to each other substantially affects the polypeptide’s overall shape. As a consequence, this system required large temperature (and bias weight) to ensure sufficient global conformational flexibility and stably reach a conformation near the target.

Though a basic trend should be maintained, these findings cannot be directly translated to other protein systems. The optimal combination of temperature *T* and bias weight *k*_*χ*_ depends on the individual system and should be determined by grid search or other systematic parameter optimization methods. In SBMs, the overall contact and dihedral energy is set equal to the number of atoms in the system [49]. This choice yields folding temperatures near 1 in the structure-based reduced units, corresponding to approximately 120 reduced GROMACS temperature units, and ensures a consistent parameterization. Thus, model-inherent absolute energies are highly system-specific and not comparable among different systems. Not only differ biomolecular systems in general and thus their respective absolute energies, but also the nature of their individual conformational transitions each associated with a specific energy barrier of different (unknown) height. Due to the high diversity among biomolecular systems, different systems require different bias weights and temperatures to suitably impact the underlying structure-based potential and provide sufficient thermal energy to induce or accelerate the conformational transition of interest. These parameters are not transferable and have to be determined separately for each system.

### Adenylate kinase

**Fig 6:**
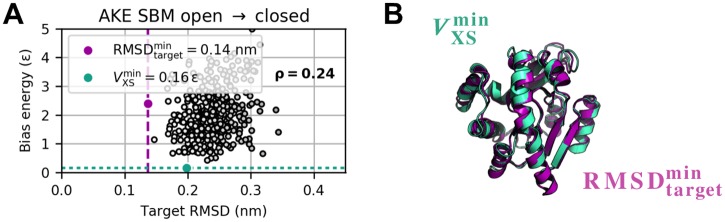
Bias potential versus target RMSD for AKE’s open-to-closed transition. (A) Bias potential versus target RMSD. (B) ${\text{RMSD}}_{\text{target}}^{\text{min}}$ (purple) and $V_{\text{XS}}^{\text{min}}$ (turquoise) structure feature a C_α_ RMSD of 0.14 nm with respect to each other.

**Fig 7:**
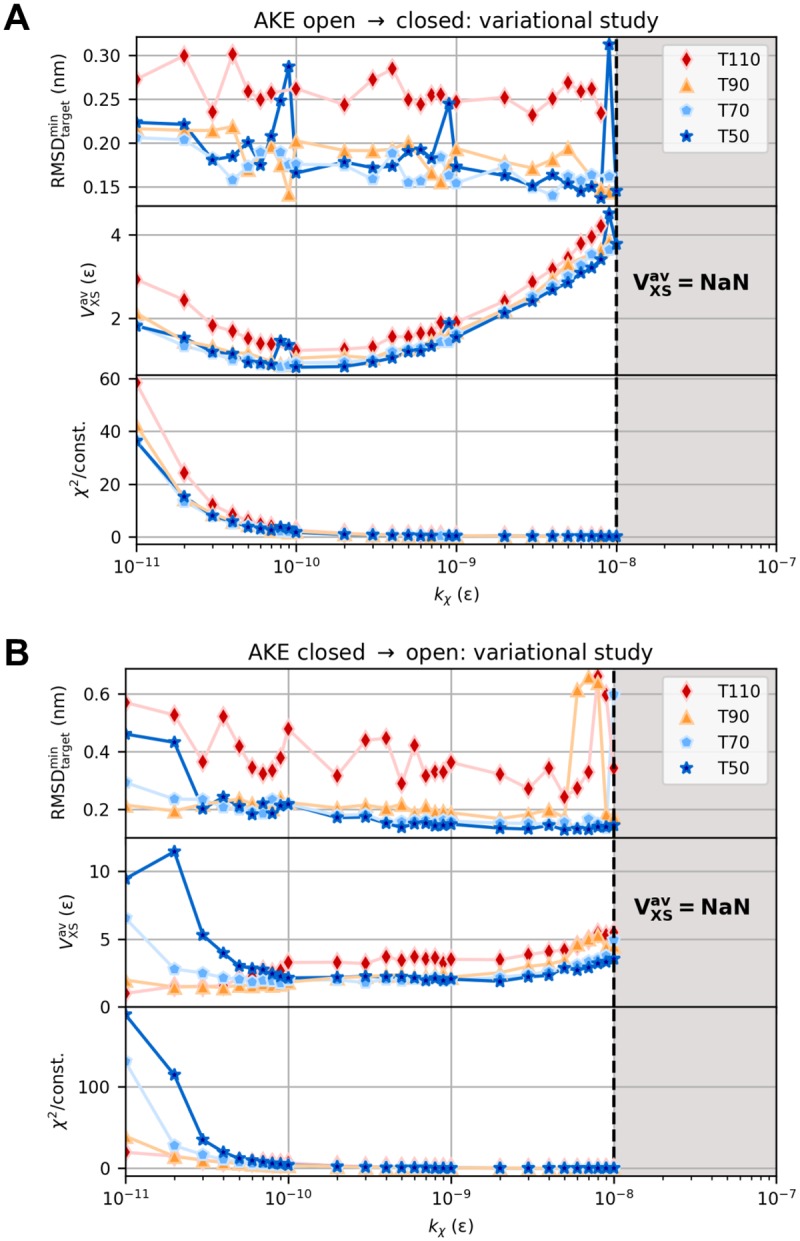
Variational study for AKE. Minimum target RMSD, average bias energy, and average *χ*^2^ dissimilarity for open-to-closed (A) and closed-to-open (B) transition as a function of coupling strength *k*_*χ*_ at different temperatures *T*. The variational series comprised 296 simulations in total.

Modeling the large-scale structural transition between open and closed conformation based on artificial difference data gives a theoretically constructed test example of a real protein movement. Simulations started from open and closed state and aimed at closed and open state, respectively. We computed artificial target difference data (Fig 2B) using the Debye equation on amino-acid level. RMSD and bias energy curves of open-to-closed and closed-to-open transition are shown in Fig 2C and Panel A in S7 Fig, respectively, illustrating how the structural similarity to initial and target state develops over the course of the simulations. Both refinements showed one clear transition from initial to target conformation in form of an immediate intercept of RMSD curves. *V*_XS_ instantaneously minimized accordingly. Subsequently, the target RMSD curves proceeded near respective average free RMSD values. Best structures as measured by target RMSD and bias energy are shown in Fig 2D and Panel B in S7 Fig, respectively. Minimum target RMSD is 0.14 nm for both directions of the conformational transition. Detailed results of analogous explicit-solvent MD simulations can be found in S8 and S9 Figs. All result parameters are summarized in Table 2. Considering computation times $\tau_{0.2}^{\text{comp}}$, structure-based refinements turned out to be faster by almost two orders of magnitude than the full-MD approach, while yielding more accurate structures in terms of minimum target RMSD. We analyzed the bias energy as a function of target RMSD (Fig 6A and Panel C in S7 Fig), which revealed positive Pearson correlations throughout. As a result of the almost instantaneous structural transitions, numerous similar conformations with small bias energy and target RMSD less than 0.2 nm do exist, yielding a dense cluster of fluctuating points (RMSD_target_, *V*_XS_) in this area. This behavior disrupts a potential linear relationship between these quantities as assumed in the Pearson correlation analysis, causing rather small but certainly positive values for *ρ*. We conducted grid-search variational studies for both open-to-closed (see Fig 7A) and closed-to-open (see Fig 7B) transition. As indicated by the regions of undefined bias potential in Fig 7, simulations using a bias weight *k*_*χ*_ greater than 10^−8^ *ε* apparently blew up. Depending on *χ*^2^, an immoderate bias weight may produce a very large bias potential. This generates an unacceptably large force, which eventually results in a failure of the integrator. For both directions of the conformational transition, the turnaround bias weight $k_{\chi }^{*}$ is in the order of 10^−10^ *ε*. At this point, the bias potential clearly exhibits its global minimum and average *χ*^2^ dissimilarity significantly drops down accordingly. In contrast to the VHP polypeptide, lower temperatures were sufficient to stably sample conformations near the target structure. This is due to the fact that the structural transition of AKE does not induce a drastic overall change in its molecular shape.

**Table 2:** Result parameters for scattering-guided simulations of AKE.

method	SBM		MD	
transition	o → c	c → o	o → c	c → o
*k*_*χ*_	8 ⋅ 10^−9^ *ε*		5 ⋅ 10^−10^ kJ/mol	
*T*	50		300 K	
${\text{RMSD}}_{\text{target}}^{\text{min}}\;\left(\text{nm}\right)$	0.14	0.14	0.18	0.16
RMSD_initial_(nm)	0.70	0.70	0.64	0.71
${\text{RMSD}}_{\text{free}}^{\text{av}}\;\left(\text{nm}\right)$	0.09	0.16	0.24	0.63
$\tau_{0.2}^{\text{comp}}\;\left(\text{hh}:\text{mm}:\text{ss}\right)$	00: 06: 40	00: 52: 25	04: 39: 15	14: 52: 20
$V_{\text{XS}}^{\text{av}}$	3.40 *ε*	3.18 *ε*	19.78 kJ/mol	8.83 kJ/mol
$V_{\text{XS}}^{\text{min}}$	0.16 *ε*	0.03 *ε*	0.47 kJ/mol	1.18 kJ/mol
${\text{RMSD}}_{\text{target}}\left(V_{\text{XS}}^{\text{min}}\right)\;\left(\text{nm}\right)$	0.20	0.24	0.21	0.26
$\text{RMSD}\left({\text{RMSD}}_{\text{target}}^{\text{min}}\;\text{-}\;V_{\text{XS}}^{\text{min}}\right)\;\left(\text{nm}\right)$	0.14	0.22	0.11	0.29

Open-to-closed and closed-to-open transition are referred to as o → c and c → o, respectively. Minimum target RMSD (${\text{RMSD}}_{\text{target}}^{\text{min}}$), associated initial RMSD (RMSD_initial_), average RMSD from a corresponding free simulation of each target (${\text{RMSD}}_{\text{free}}^{\text{av}}$), computation time associated with a target RMSD less than 0.2 nm ($\tau_{0.2}^{\text{comp}}$), average bias potential ($V_{\text{XS}}^{\text{av}}$), minimum bias potential ($V_{\text{XS}}^{\text{min}}$), target RMSD associated with minimum bias energy (${\text{RMSD}}_{\text{target}}\left(V_{\text{XS}}^{\text{min}}\right)$), and the RMSD between minimum target RMSD structure and minimum bias energy structure ($\text{RMSD}({\text{RMSD}}_{\text{target}}^{\text{min}}\;\text{-}\;V_{\text{XS}}^{\text{min}})$) are listed.

### Lysine-arginine-ornithine binding protein

Upon binding lysine, LAO protein (Fig 3A) experiences major structural change [56]. Modeling this domain motion based on artificial difference data gives another test case of a real protein movement. Starting from the crystal structure of the unliganded holo state, these simulations aimed at the unbound apo state and vice versa. Reference and target scattering were calculated from the crystal structures with CRYSOL [57] and thus implicitly include hydration shell contributions. We generated artificial difference data (Fig 3B) by subtracting the initial solution scattering from the target solution scattering. Time-dependent initial and target RMSD as well as bias potential are shown in Fig 3C and Panel A in S10 Fig for structure-based holo-to-apo and apo-to-holo simulations, respectively. Biasing simulations towards theoretical difference data resulted in the transitions to readily occur. The bias potential minimized almost instantaneously according to the trajectory’s convergence towards the target state. The final target RMSD of approx. 0.2 nm was consistent with corresponding free simulations. For both directions of the conformational transition, best structures exhibit a minimum target RMSD of 0.09 nm (Fig 3D and Panel B in S10 Fig). Structure-based refinements were capable of producing structures in full agreement with the target state. Provided equal computing resources, they required only a small fraction of computing time by comparison with analogous explicit-solvent MD simulations. Detailed full-MD results can be found in S11 and S12 Figs. Considering the holo-to-apo transition, the explicit-solvent refinement (*t*_sim_ = 10 ns) lasted for 4 d 15 h 5 min 35 s, whereas the SBM run (*t*_sim_ = 2000 arb) spanned 4 h 11 min 55 s. According to computation times $\tau_{0.2}^{\text{comp}}$ related to the trajectory approaching a state with a target RMSD less than 0.2 nm, the SBM proved to be up to ten times faster in terms of wall-clock time. All result parameters are summarized in Table 3.

**Table 3:** Result parameters for scattering-guided simulations of LAO protein.

method	SBM			MD	
*k*_*χ*_	9 ⋅ 10^−11^ *ε*			1 ⋅ 10^−9^ kJ/mol	
*T*	50			300 K	
transition	h → a		a → h	h → a	a → h
SAXS data	clean	noisy	clean	clean	
${\text{RMSD}}_{\text{target}}^{\text{min}}\;\left(\text{nm}\right)$	0.09	0.09	0.09	0.09	0.07
RMSD_initial_(nm)	0.46	0.51	0.47	0.48	0.49
${\text{RMSD}}_{\text{free}}^{\text{av}}\;\left(\text{nm}\right)$	0.08		0.12	0.20	0.18(0.28)
$\tau_{0.2}^{\text{comp}}\;\left(\text{hh}:\text{mm}:\text{ss}\right)$	00: 12: 05	00: 03: 30	00: 01: 55	01: 32: 50	00: 47: 20
$V_{\text{XS}}^{\text{av}}$	3.36 *ε*	48.44 *ε*	3.94 *ε*	24.36 kJ/mol	69.06 kJ/mol
$V_{\text{XS}}^{\text{min}}$	1.26 *ε*	47.93 *ε*	1.99 *ε*	14.17 kJ/mol	38.41 kJ/mol
${\text{RMSD}}_{\text{target}}\left(V_{\text{XS}}^{\text{min}}\right)\;\left(\text{nm}\right)$	0.18	0.25	0.14	0.16	0.12
$\text{RMSD}\left({\text{RMSD}}_{\text{target}}^{\text{min}}\;\text{-}\;V_{\text{XS}}^{\text{min}}\right)\;\left(\text{nm}\right)$	0.16	0.25	0.13	0.14	0.11

Holo-to-apo and apo-to-holo transition are referred to as h → a and a → h, respectively. Minimum target RMSD (${\text{RMSD}}_{\text{target}}^{\text{min}}$), associated initial RMSD (RMSD_initial_), average RMSD from a corresponding free simulation of each target (${\text{RMSD}}_{\text{free}}^{\text{av}}$), computation time associated with a target RMSD less than 0.2 nm ($\tau_{0.2}^{\text{comp}}$), average bias potential ($V_{\text{XS}}^{\text{av}}$), minimum bias potential ($V_{\text{XS}}^{\text{min}}$), target RMSD associated with minimum bias energy (${\text{RMSD}}_{\text{target}}\left(V_{\text{XS}}^{\text{min}}\right)$), and the RMSD between minimum target RMSD structure and minimum bias energy structure ($\text{RMSD}({\text{RMSD}}_{\text{target}}^{\text{min}}\;\text{-}\;V_{\text{XS}}^{\text{min}})$) are listed.

**Fig 8:**
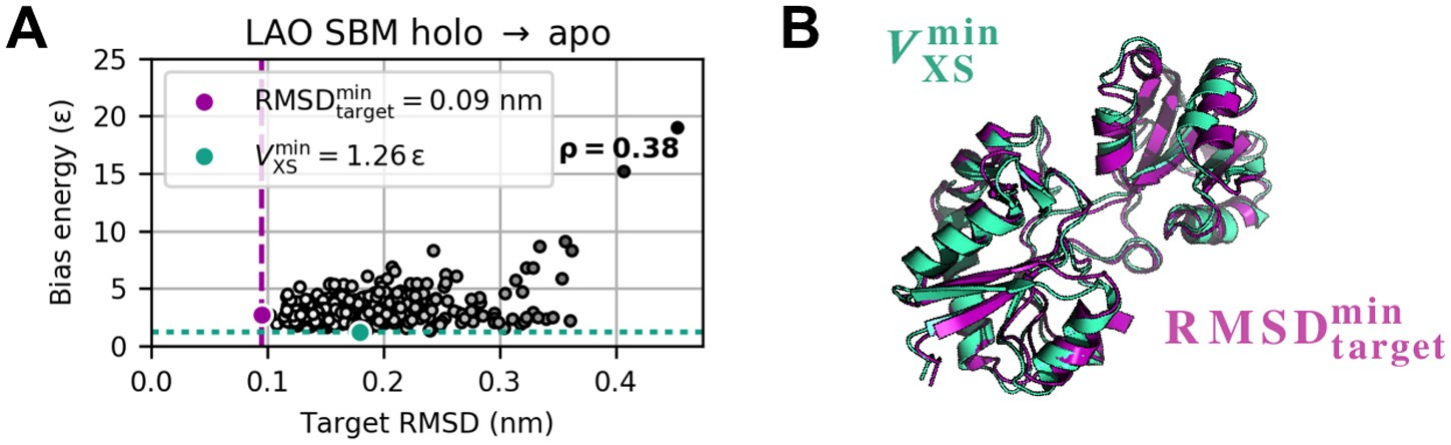
Bias potential versus target RMSD for LAO protein’s holo-to-apo transition. (A) Bias potential versus target RMSD. (B) ${\text{RMSD}}_{\text{target}}^{\text{min}}$ (purple) and $V_{\text{XS}}^{\text{min}}$ (turquoise) structure feature a C_α_ RMSD of 0.16 nm with respect to each other.

As in the other test systems, bias energy and target RMSD exhibit positive Pearson correlations throughout. According to Fig 8A and Panel C in S11 Fig, the structural diversity at equal bias potential levels is similar for SBM and explicit-solvent MD. Though the best structure cannot definitely be identified from a trajectory on the basis of *V*_XS_ on its own, the bias potential can serve as a primary indicator for a simulation’s current state and eventual success or failure.

**Fig 9:**
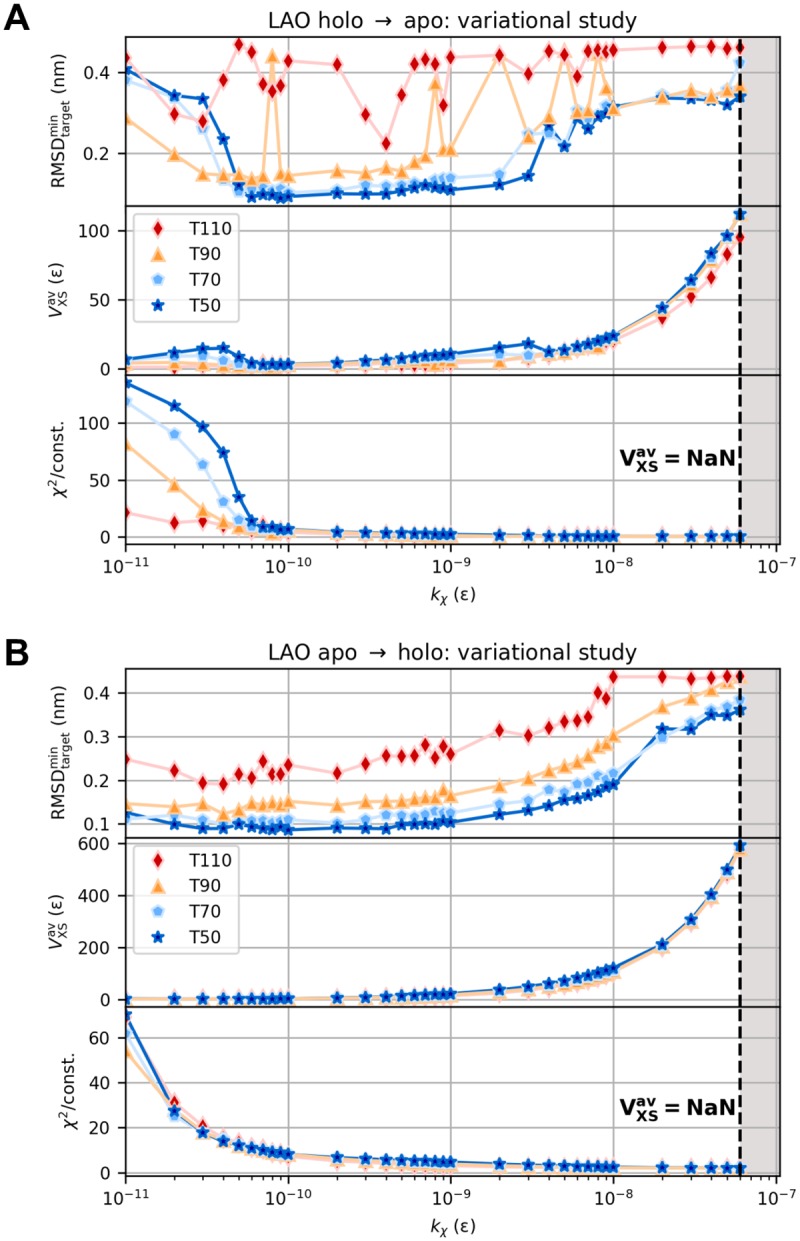
Variational study for LAO protein. Minimum target RMSD, average bias energy, and average *χ*^2^ dissimilarity for holo-to-apo (A) and apo-to-holo (B) transition as a function of coupling strength *k*_*χ*_ at different temperatures *T*. The variational series comprised 296 simulations in total.

**Fig 10:**
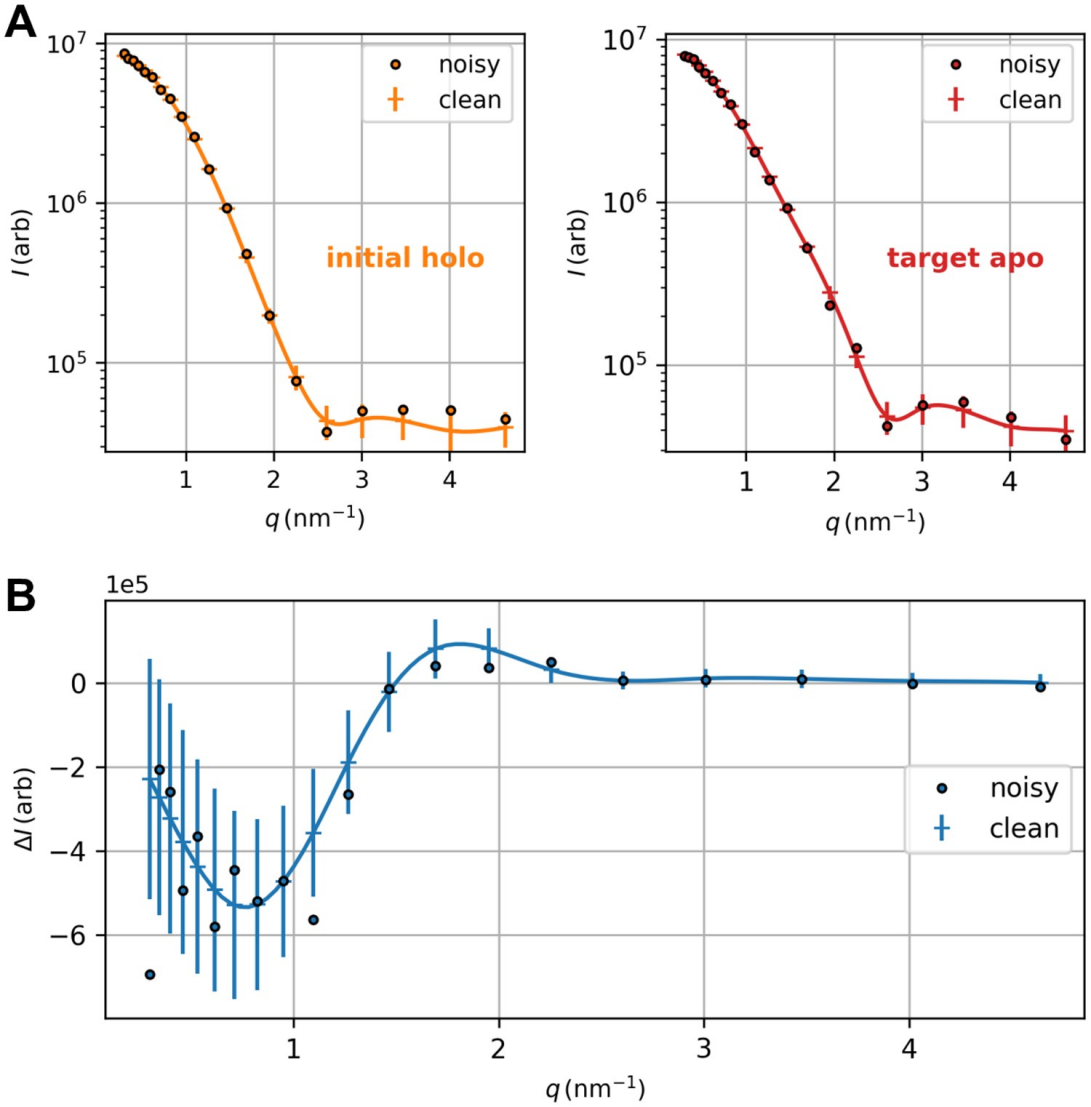
Noisy artificial difference data for LAO protein’s holo-to-apo transition. (A) Blurred absolute intensities of initial holo state (orange circles, left) and target apo state (red circles, right) along with related clean scattering curves (solid lines) and error bars. For each *q* point, a ‘blurred’ intensity value was randomly taken from a Gaussian distribution centered at the corresponding clean intensity. The standard deviation and intensity error was calculated as the square root of the clean intensity value. (B) Noisy difference scattering data (blue circles) were obtained by subtracting the blurred absolute initial intensity (orange circles above) from the blurred absolute target intensity (red circles above). Clean difference data are depicted as solid line with related error bars. Errors were derived via usual error propagation and used to individually weight the different *q* points in the simulation.

Grid-search variational studies for both holo-to-apo and apo-to-holo transition revealed a similar behavior as for AKE. As highlighted by the regions of undefined bias potential in Fig 9, simulations applying a bias weight *k*_*χ*_ greater than 6 ⋅ 10^−8^ *ε* failed due to excessively large scattering-related forces. For both directions of the structural transition, the turnaround bias weight $k_{\chi }^{*}$ is approx. 10^−10^ *ε*. Average *χ*^2^ dissimilarity clearly minimizes here (Fig 9A and 9B, bottom), whereas the bias potential does not have a distinct minimum as is the case for AKE but starts to monotonically increase as a function of *k*_*χ*_ (Fig 9A and 9B, middle). Again, the evolution of minimum target RMSD indicates lower temperatures to be sufficient to stably reach the target conformation (Fig 9A and 9B, top). This is due to the fact that the conformational transition corresponds to a relative movement of subdomains in the structure so that the molecular shape does not experience a drastic overall change. To assess the method’s robustness towards errors in the scattering data, we conducted a structure-based refinement of LAO protein’s holo-to-apo transition towards noisy artificial difference data. Theoretical absolute scattering curves of reference and target structure were blurred according to a random Gaussian noise. For each *q* point, mean and standard deviation were modeled as the related clean intensity value and its square root, respectively. Details are described in S3 Appendix. We calculated noisy difference data by subtracting the blurred reference intensity from the blurred target intensity (Fig 10). Using usual error propagation, errors were calculated as the sum of the Gaussians’ absolute standard deviations and used to individually weight the *q* points in the simulation. We applied the same parameters as in the refinement towards clean data. Although the bias potential levels off at a considerably higher value, which is to be expected, the simulation could produce equal-quality structures and thus proved to be robust against errors, at least for the level of noise assumed here (see S13 Fig). Note once more that scattering-guided SBM simulations dispense with computationally expensive solvent effects. In view of these results, we did not find a need for explicit solvation in refinement simulations comprising small-angle scattering data up to a maximum momentum transfer of 5 nm^−1^. The fact that SBM simulations coupled to small-angle difference scattering data could reproduce each target state with high accuracy indicates that such curves hold sufficient information to guide the simulation towards the correct conformation, at least for the systems studied here. Regardless of their reduced level of complexity by comparison with explicit-solvent MD, scattering-guided SBM simulations produced equal-quality results in a small fraction of computing time.

### Conclusions

A fundamental paradigm in protein biophysics is the interdependency of macromolecular structure and function. In light of this, small-angle X-ray scattering has significantly gained in importance, especially for structural analyses of dissolved macromolecules. Accurate interpretation of resulting scattering intensities in terms of atomistic models is still a challenging task. By incorporating information from SAXS into structure-based models, we aimed at efficiently interpreting scattering data within computational simulations. Studying three different test systems, we have proven our method to be capable of effectively probing real protein transitions, based only on low-resolution scattering data. Giving results equivalent to those from analogous full-MD methods [20], scattering-guided SBM simulations could expedite interpretation of intensities from biological SAXS by about two orders of magnitude. Such simulations benefit from extensive sampling as a result of their intrinsically accelerated dynamics. They could rapidly generate structural ensembles in accordance with the input data and provide a valuable alternative for efficient refinement of atomistic structures against SAXS data. Thus, they are particularly suitable for initial high-throughput analyses and can easily perform on usual commodity hardware. If desired, the resultant structure can still be given a final polish within a regular MD force field. As a result of technical advances in light sources and detectors, the wide-angle regime encoding local structural fluctuations has become increasingly accessible in the experiment. So as to level up with experimental resolution, increasingly fine-grained modeling may then be indicated at the cost of leaping computational demands [5, 21].

Finally, it is important to note that some systems cannot be analyzed straightforward using SAXS. In all test systems, structural transitions could be modeled by a collective movement along one effective degree of freedom, which influences the protein’s shape and thus the difference curve at *q* ≤ 2 nm^−1^ crucially. As a consequence, structural fits were unique. However, at higher *q* values, multiple candidate structures can generate interfering features in the difference profiles. For example, the structural change in the cytoplasmic portion of a sensor histidine kinase protein (PDB code 2C2A [58]) induces a C_α_ RMSD shift of 1.25 nm. This conformational transition can effectively be described as a rotation of one subdomain around a helix bundle, but influences the overall molecular shape only marginally. Despite a substantial decrease in bias potential, refinements towards theoretical difference data did not converge to the target structure. This implies that structures exist, which adequately reproduce the difference data, but are not compatible with structural models obtained from crystallographic methods. These findings are in accordance with results presented in Ref [20] and due to a lack of information in the low-resolution experimental data, resulting in unaccomplishably high demands on the theoretical model.

Having said this, the protocol for interpretation of SAXS data within SBMs established in this work can serve as a suitable starting point for further developments. These include e.g. expanding single-basin SBMs to multi-Gō models with several minima and testing other functional forms of the bias potential. Furthermore, we intend to directly interface the structure-based refinement framework with parameter optimization methods such as Bayesian inference. In addition, we see several possibilities to extend our hybrid framework to additionally account for information derived from other experimental techniques than SAXS. We plan to extend the framework by considering co-evolutionary contact information from biomolecular sequence data [36], distance and angle information from NMR spectroscopy, and cryo-EM density maps [6]. Whereas co-evolutionary information can be considered by additional potential terms similar to usual SBM native contacts, NMR distance and angle information can be accounted for by implementing suitable spatial restraints. Provided cryo-EM data, another energetic term can be introduced to bias the structure towards the electron density map based on a spatial overlap. Performing simulations with such a hybrid structure-based/biased/restraint force field, the system can relax into configurations that are consistent with all these contributions.

## Materials and methods

All simulations were carried out on a standard desktop PC with an Intel Core i7-6700 CPU comprising eight cores at a frequency of 3.40 GHz. We used a version of GROMACS 5 modified by the scattering-guided MD extension [20, 59] and molecular dynamics parameters listed in S4 Appendix. Simulations differed only with respect to their couplings *k*_*χ*_ to the scattering data and reduced GROMACS temperatures *T*. As information on crucial structural features, i.e. molecular shape and global conformational changes, is contained in the small-angle regime, only *q* values up to a maximum of 5 nm^−1^ were included. We used theoretical scattering data calculated from pure initial and final states for method validation. In a SAXS experiment, the measured intensity pattern might reflect a linear combination of scattering intensities from a mixture of conformations in the sample. However, starting from the pure initial state, conformational transitions were assumed to take place entirely in the simulations. This means, in a corresponding experiment, all protein molecules would undergo the structural transition of interest from initial to final state. Consequently, *α* was set to 1 in all simulations.

### Set-up of scattering-guided SBM simulations

As a starting point, all-atom SBMs were constructed from the considered system’s initial structure with eSBMTools [30] to obtain suitable coordinate and topology files. Debye scattering terms are encoded as a special type of bonded interaction in the topology file [20]. Scattering topology as well as related extended coordinate file were constructed with gmx genrestr. This command creates half a matrix of virtual-site type-3 pairs, i.e. Debye terms, for the input coordinate file. Amino-acid scatterers centered on virtual interaction sites at the respective residue’s center of mass were used. All residues were considered. The resulting topology include file was added to the system’s topology directly after the atoms section. The corresponding atom type ‘MW’ was manually appended to the atom types table. If Debye scattering data were used as a target, the initial scattering was calculated using gmx waxsdebye. Suitably adjusted run parameters for the SBM refinement are listed in S4 Appendix. Temperatures *T* and bias weights *k*_*χ*_ were set as described in Results and discussion. Finally, SBMs were preprocessed with gmx grompp and run with gmx mdrun.

### Set-up of scattering-guided full-MD simulations

The set-up of explicit-solvent MD simulations followed the common steps of adding hydrogen atoms, choosing potential and water model, neutralizing electric charge by adding an appropriate number of ions, minimizing energy, and equilibrating temperature and pressure. We used the CHARMM27 force field [60], TIP3P water model [61], Verlet cut-off scheme, and a constant temperature of 300 K. Electrostatics were treated with the Particle Mesh Ewald method. Parrinello-Rahman pressure coupling and V-rescale temperature coupling were applied. To obtain coordinate and topology file, initial models were preprocessed and protonated with gmx pdb2gmx. A periodic cubic box exceeding twice the longest inter-protein distance was constructed with gmx editconf. The structure was initially energy-minimized using the GROMACS preprocessor gmx grompp and simulation command gmx mdrun. After solvation and electric-charge neutralization, the structure was energy-minimized again. Subsequently, systems were equilibrated in the canonical and isothermal-isobaric ensemble until temperature and pressure converged. Non-hydrogen atoms were position-restrained to their initial positions. A half-matrix of Debye terms was constructed with gmx genrestr for the NPT-equilibrated structure, including all residues and using amino-acid scatterers. This created the scattering topology, which was manually included into the system’s topology. The initial reference scattering was generated with gmx waxsdebye. After preprocessing with gmx grompp, the scattering-guided MD simulation was performed using the gmx mdrun command. Results are shown for coupling strengths *k*_*χ*_ optimized via grid-search variational studies comprising 16 simulations in total for each system.

## Supporting information

S1 Appendix Theory on molecular solution X-ray scattering. (PDF)

S2 Appendix Radius of gyration and asphericity analysis. Asphericity was calculated according to the definition in [62].
(PDF)

S3 Appendix Modeling noise in the target scattering data. (PDF)

S4 Appendix Molecular dynamics parameter file for scattering-guided SBM simulations. (PDF)

S5 Appendix How-to tutorial. The tutorial requires valid installations of the scattering-guided GROMACS software [20], the python package eSBMTools [30], and the molecular visualization program VMD [63].
(PDF)

S1 Fig Radius of gyration and asphericity for VHP polypeptide. Shape parameters for VHP elongated-to-bent and bent-to-elongated transition (A and B, respectively). Radius of gyration (red) and asphericity (blue) versus simulated time are shown at the top and bottom of each panel. Results at the left and right of each panel belong to scattering-guided full-MD and SBM simulations, respectively.
(TIF)

S2 Fig Radius of gyration and asphericity for AKE. Shape parameters for AKE open-to-closed and closed-to-open transition (A and B, respectively). Radius of gyration (red) and asphericity (blue) versus simulated time are shown at the top and bottom of each panel. Results at the left and right of each panel belong to scattering-guided full-MD and SBM simulations, respectively.
(TIF)

S3 Fig Radius of gyration and asphericity for LAO protein. Shape parameters for LAO holo-to-apo and apo-to-holo transition (A and B, respectively). Radius of gyration (red) and asphericity (blue) versus simulated time are shown at the top and bottom of each panel. Results at the left and right of each panel belong to scattering-guided full-MD and SBM simulations, respectively.
(TIF)

S4 FigSBM simulation of VHP bent-to-elongated transition.Results are shown for parameters (*T*, *k*_*χ*_) = (90, 5 ⋅ 10^−8^ *ε*). (A) Initial and target RMSD (top) and bias energy (bottom) versus simulated time. (B) Best structures as measured by target RMSD and bias energy. ${\text{RMSD}}_{\text{target}}^{\text{min}}$ structure (purple) and $V_{\text{XS}}^{\text{min}}$ structure (turquoise) feature target RMSDs of 0.19 nm and 0.37 nm, respectively. (C) Bias potential versus target RMSD. (D) ${\text{RMSD}}_{\text{target}}^{\text{min}}$ (purple) and $V_{\text{XS}}^{\text{min}}$ (turquoise) structure exhibit an RMSD of 0.31 nm with respect to each other.(TIF)

S5 FigExplicit-solvent MD simulation of VHP elongated-to-bent transition.Results are shown for parameters (*T*, *k*_*χ*_) = (330 K, 5 ⋅ 10^−9^ kJ/mol). (A) Initial and target RMSD (top) and bias energy (bottom) versus simulated time. (B) Best structures as measured by target RMSD and bias energy. ${\text{RMSD}}_{\text{target}}^{\text{min}}$ structure (purple) and $V_{\text{XS}}^{\text{min}}$ structure (turquoise) feature target RMSDs of 0.08 nm and 0.24 nm, respectively. (C) Bias potential versus target RMSD. (D) ${\text{RMSD}}_{\text{target}}^{\text{min}}$ (purple) and $V_{\text{XS}}^{\text{min}}$ (turquoise) structure exhibit an RMSD of 0.21 nm with respect to each other.(TIF)

S6 FigExplicit-solvent MD simulation of VHP bent-to-elongated transition.Results are shown for parameters (*T*, *k*_*χ*_) = (330 K, 5 ⋅ 10^−9^ kJ/mol). (A) Initial and target RMSD (top) and bias energy (bottom) versus simulated time. Apparently, the simulation could only selectively sample conformations near the target structure. (B) Best structures as measured by target RMSD and bias energy. ${\text{RMSD}}_{\text{target}}^{\text{min}}$ structure (purple) and $V_{\text{XS}}^{\text{min}}$ structure (turquoise) feature target RMSDs of 0.25 nm and 0.39 nm, respectively. (C) Bias potential versus target RMSD. (D) ${\text{RMSD}}_{\text{target}}^{\text{min}}$ (purple) and $V_{\text{XS}}^{\text{min}}$ (turquoise) structure exhibit an RMSD of 0.28 nm with respect to each other.(TIF)

S7 FigSBM simulation of AKE closed-to-open transition.Results are shown for parameters (*T*, *k*_*χ*_) = (50, 8 ⋅ 10^−9^ *ε*). (A) Initial and target RMSD (top) and bias energy (bottom) versus simulated time. (B) Best structures as measured by target RMSD and bias energy. ${\text{RMSD}}_{\text{target}}^{\text{min}}$ structure (purple) and $V_{\text{XS}}^{\text{min}}$ structure (turquoise) have target RMSDs of 0.14 nm and 0.24 nm, respectively. (C) Bias potential versus target RMSD. (D) ${\text{RMSD}}_{\text{target}}^{\text{min}}$ (purple) and $V_{\text{XS}}^{\text{min}}$ (turquoise) structure exhibit an RMSD of 0.22 nm with respect to each other.(TIF)

S8 FigExplicit-solvent MD simulation of AKE open-to-closed transition.Results are shown for parameters (*T*, *k*_*χ*_) = (300 K, 5 ⋅ 10^−10^ kJ/mol). (A) Initial and target RMSD (top) and bias energy (bottom) versus simulated time. (B) Best structures as measured by target RMSD and bias energy. ${\text{RMSD}}_{\text{target}}^{\text{min}}$ structure (purple) and $V_{\text{XS}}^{\text{min}}$ structure (turquoise) have target RMSDs of 0.18 nm and 0.21 nm, respectively. (C) Bias potential versus target RMSD. (D) ${\text{RMSD}}_{\text{target}}^{\text{min}}$ (purple) and $V_{\text{XS}}^{\text{min}}$ (turquoise) structure exhibit an RMSD of 0.11 nm with respect to each other.(TIF)

S9 FigExplicit-solvent MD simulation of AKE closed-to-open transition.Results are shown for parameters (*T*, *k*_*χ*_) = (300 K, 5 ⋅ 10^−10^ kJ/mol). (A) Initial and target RMSD (top) and bias energy (bottom) versus simulated time. (B) Best structures as measured by target RMSD and bias energy. ${\text{RMSD}}_{\text{target}}^{\text{min}}$ structure (purple) and $V_{\text{XS}}^{\text{min}}$ structure (turquoise) have target RMSDs of 0.16 nm and 0.26 nm, respectively. (C) Bias potential versus target RMSD. (D) ${\text{RMSD}}_{\text{target}}^{\text{min}}$ (purple) and $V_{\text{XS}}^{\text{min}}$ (turquoise) structure exhibit an RMSD of 0.29 nm with respect to each other.(TIF)

S10 FigSBM simulation of LAO apo-to-holo transition.Results are shown for parameters (*T*, *k*_*χ*_) = (50, 9 ⋅ 10^−11^ *ε*). (A) Initial and target RMSD (top) and bias energy (bottom) versus simulated time. (B) Best structures as measured by target RMSD and bias energy. ${\text{RMSD}}_{\text{target}}^{\text{min}}$ structure (purple) and $V_{\text{XS}}^{\text{min}}$ structure (turquoise) have target RMSDs of 0.09 nm and 0.14 nm, respectively. (C) Bias potential versus target RMSD. (D) ${\text{RMSD}}_{\text{target}}^{\text{min}}$ (purple) and $V_{\text{XS}}^{\text{min}}$ (turquoise) structure exhibit an RMSD of 0.13 nm with respect to each other.(TIF)

S11 FigExplicit-solvent MD simulation of LAO holo-to-apo transition.Results are shown for parameters (*T*, *k*_*χ*_) = (300 K, 1 ⋅ 10^−9^ kJ/mol). (A) Initial and target RMSD (top) and bias energy (bottom) versus simulated time. (B) Best structures as measured by target RMSD and bias energy. ${\text{RMSD}}_{\text{target}}^{\text{min}}$ structure (purple) and $V_{\text{XS}}^{\text{min}}$ structure (turquoise) have target RMSDs of 0.09 nm and 0.16 nm, respectively. (C) Bias potential versus target RMSD. (D) ${\text{RMSD}}_{\text{target}}^{\text{min}}$ (purple) and $V_{\text{XS}}^{\text{min}}$ (turquoise) structure exhibit an RMSD of 0.14 nm with respect to each other.(TIF)

S12 FigExplicit-solvent MD simulation of LAO apo-to-holo transition.Results are shown for parameters (*T*, *k*_*χ*_) = (300 K, 1 ⋅ 10^−9^ kJ/mol). (A) Initial and target RMSD (top) and bias energy (bottom) versus simulated time. (B) Best structures as measured by target RMSD and bias energy. ${\text{RMSD}}_{\text{target}}^{\text{min}}$ structure (purple) and $V_{\text{XS}}^{\text{min}}$ structure (turquoise) have target RMSDs of 0.07 nm and 0.12 nm, respectively. (C) Bias potential versus target RMSD. (D) ${\text{RMSD}}_{\text{target}}^{\text{min}}$ (purple) and $V_{\text{XS}}^{\text{min}}$ (turquoise) structure exhibit an RMSD of 0.11 nm with respect to each other.(TIF)

S13 FigLAO holo-to-apo SBM simulation using noisy data.(A) Initial and target RMSD (top) and bias energy (bottom) versus simulated time. (B) Best structures as measured by target RMSD and bias energy. ${\text{RMSD}}_{\text{target}}^{\text{min}}$ structure (purple) and $V_{\text{XS}}^{\text{min}}$ structure (turquoise) have target RMSDs of 0.09 nm and 0.25 nm, respectively. (C) Bias potential versus target RMSD. (D) ${\text{RMSD}}_{\text{target}}^{\text{min}}$ (purple) and $V_{\text{XS}}^{\text{min}}$ (turquoise) structure exhibit an RMSD of 0.25 nm with respect to each other.(TIF)

S1 Archive Set-up files for scattering-guided simulations of all test systems. (ZIP)

S2 Archive Set-up files for how-to tutorial. (ZIP)
