# Supplementary material for: Rapid interpretation of small-angle X-ray scattering data
Source: PLoS Comput Biol. 2019 Mar 22;15(3):e1006900. doi: 10.1371/journal.pcbi.1006900 (PMC6447237; doi:10.1371/journal.pcbi.1006900)
Supplement: S1 Appendix — (PDF) [file pcbi.1006900.s001.pdf]

S1 Appendix Theory on molecular solution X-ray scattering.

In the following, vectors are represented by upright boldface letters, according to the common typographic convention.

Biomolecules in solution can be modeled as particles with scattering density $\rho_{\mathrm{m}}(\mathbf{r})$ surrounded by a solvent with average scattering density ρ_{s}. The molecular hydration shell is modeled by a border layer with excess density $\Delta\rho_{\mathrm{b}}$ compared to bulk solvent. Scattering density is a measure of a material's scattering power. It increases with physical density as well as intrinsic scattering power of the individual scatterers. In SAXS, X-ray scattering arises from elastic interactions of incident X-ray photons with the atoms' electrons. Scattering power thus scales with electron density, giving a measure of the probability of an electron being present at a specific location. SAXS basically resolves the difference, i.e. contrast, in electron density between the biomolecules and the solvent. In theory, the intensity $I(q)$ from a dilute sample is proportional to the spherically averaged scattering of a single molecule minus excluded-volume contributions plus a hydration shell term [47]

$$I(q) = \left\langle \left| A_{\mathrm{m}}(\mathbf{q}) - \rho_{\mathrm{s}} A_{\mathrm{s}}(\mathbf{q}) + \Delta\rho_{\mathrm{b}} A_{\mathrm{b}}(\mathbf{q}) \right|^2 \right\rangle_{\Omega}, \tag{1}$$

where the absolute momentum transfer $q = |\mathbf{q}| = 4\pi \sin\theta/\lambda$. 2θ is the scattering angle and λ the X-ray wavelength. $A_{\mathrm{m}}(\mathbf{q})$ is the scattering amplitude from the biomolecule in vacuum, $A_{\mathrm{s}}(\mathbf{q})$ from the solvent's excluded volume, and $A_{\mathrm{b}}(\mathbf{q})$ from the molecular hydration shell. $< ... >_{\Omega}$ denotes an orientational average in reciprocal space to account for the proteins' random orientations. Eq 1 provides the theoretical basis for solution scattering. For a given conformation, the scattering is contributed from (i) the actual protein in vacuum, (ii) the excluded solvent volume, and (iii) the hydration shell's relative electron density. The scattering from the protein itself can be derived as follows. Considering a molecule illuminated by a monochromatic plane wave, its atoms interacting with the incident radiation become sources of spherical waves in return. For a single scattering event by an atom at position $\mathbf{r}_j$, the amplitude $A_j(\mathbf{q})$ can be expressed as

$$A_j(\mathbf{q}) = f_j(q) \cdot \exp(i\mathbf{q}\mathbf{r}_j). \tag{2}$$

f_j is the atomic form factor, a measure of the scattering amplitude of a wave by an isolated atom. In the limit of $q \to 0$, f_j equals the atom's electron number. Within one molecule with fixed orientation in vacuum, the single amplitudes of all N atoms have to be summed up to form the overall scattering amplitude

$$\begin{aligned} A_{\mathrm{m}}(\mathbf{q}) &= \sum_{j=1}^{N} A_j(\mathbf{q}) = \sum_{j=1}^{N} f_j(q) \cdot \exp(i\mathbf{q}\mathbf{r}_j) \\ &\equiv \int_V f_{\mathrm{e}}(q)\rho_{\mathrm{m}}(\mathbf{r})\exp(i\mathbf{q}\mathbf{r})\mathrm{d}\mathbf{r} = f_{\mathrm{e}}(q) \cdot \mathfrak{F}[\rho_{\mathrm{m}}(\mathbf{r})]. \end{aligned} \tag{3}$$

The intensity is then obtained as the squared amplitude

$$I(\mathbf{q}) = |A(\mathbf{q})|^2. \tag{4}$$

In the continuum limit of Eq 3, the amplitude from an ensemble of atoms turns out to be the form-factor weighted Fourier transform $\mathfrak{F}[\rho_{\mathrm{m}}(\mathbf{r})]$ of the sample's electron density. Assuming the Born approximation, the corresponding form factor $f_{\mathrm{e}}(q)$ is the Fourier transform of the molecule's electric charge distribution normalized to its overall charge. For spatially isotropic particle distributions as in SAXS, the measured intensity pattern

constitutes a rotational average in reciprocal space, representing the multitude of distinct molecular orientations in the sample. This yields the well-known Debye formula, which states spherically averaged intensity of a molecule described as discrete sum of elementary scatterers to be [44]

$$I(q) = \langle I(\mathbf{q}) \rangle_\Omega = \sum_{i,j} f_i(q)\, f_j(q)\, \mathrm{sinc}\,(q r_{ij}), \tag{5}$$

where r_{ij} are the interatomic distances. The Debye equation as it stands does not account for any solvent contributions. With biomolecules not being available in vacuum but in dilute solution, SAXS is a contrast method, where the scattering signal is generated from a difference in average electron densities of the molecules considered and the bulk solvent. Thus, one principally has to account for solvent contributions to the measured scattering intensities. Excluded solvent can be included by a modification of atomic scattering factors, assigning a Gaussian sphere to all atoms [45, 47]

$$f'_i(q) = f_i(q) - \nu_i \rho_\mathrm{s} \exp\left(-\pi \nu_i^{2/3} q^2\right) \tag{6}$$

ν_i are the experimentally determined tabulated volumes of each atom. Thus, the scattering from the protein considering excluded-volume effects reads [47]

$$I(q) = \left\langle |A_\mathrm{m}(\mathbf{q}) - \rho_\mathrm{s} A_\mathrm{s}(\mathbf{q})|^2 \right\rangle_\Omega = \sum_{ij} f'_i(q)\, f'_j(q)\, \mathrm{sinc}\,(q r_{ij}). \tag{7}$$

Furthermore, scattering is contributed from the relative electron density of the protein-surrounding primary hydration layer to the extent that it differs from bulk solvent. In calculations using implicit water models, the solvent is modeled as a continuous electron density. The hydration layer is considered by a homogeneous excess electron density, which is typically 10% to 15% of the bulk solvent density [57]. Alternatively, it can be included by further modifying the form factors. These procedures include a system-specific free parameter, that is not easily accessible and thus usually adjusted by fitting the calculated to an experimental scattering curve. Consequently, the validity of such an implicit representation of the solvation shell's structure remains uncertain [47].
