# Supplementary material for: Rapid interpretation of small-angle X-ray scattering data
Source: PLoS Comput Biol. 2019 Mar 22;15(3):e1006900. doi: 10.1371/journal.pcbi.1006900 (PMC6447237; doi:10.1371/journal.pcbi.1006900)
Supplement: S2 Appendix — Asphericity was calculated according to the definition in [62]. (PDF) [file pcbi.1006900.s002.pdf]

S2 Appendix Radius of gyration and asphericity analysis.
To ensure structural congruence between the ensembles generated by structure-based and explicit-solvent MD simulations, we calculated both radius of gyration R_g and asphericity Δ as functions of simulated time. The gyration tensor T of a molecule consisting of N (spherical) atoms at Cartesian positions $\mathbf{r}_i$, $i = 1, ..., N$, can be expressed as [62]

$$T_{\alpha\beta} = \frac{1}{2N^2} \cdot \sum_{i,j=1}^{N} (r_{i\alpha} - r_{j\alpha}) \cdot (r_{i\beta} - r_{j\beta}), \tag{1}$$

where $r_{i\alpha}$ is the αth component of $\mathbf{r}_i$ and α, β are x, y, and z. Asphericity was calculated as [62]

$$\Delta = \frac{3 \sum\limits_{i=1}^{3} (\lambda_i - \bar{\lambda})^2}{2(\mathrm{tr}\, T)^2}, \tag{2}$$

where λ_i are the eigenvalues of T with $\mathrm{tr}\, T = \sum_i \lambda_i = R_\mathrm{g}^2$ and $\bar{\lambda} = \mathrm{tr}\, T/3$. We computed λ_i using the `GROMACS` tool `gmx polystat` [59]. R_g was extracted from the trajectories using `gmx gyrate` [59]. As indicated in S1 Fig, S2 Fig, and S3 Fig, both shape parameters confirmed the structural conformity of ensembles generated by SBM and explicit-solvent MD.
