# Supplementary material for: Rapid interpretation of small-angle X-ray scattering data
Source: PLoS Comput Biol. 2019 Mar 22;15(3):e1006900. doi: 10.1371/journal.pcbi.1006900 (PMC6447237; doi:10.1371/journal.pcbi.1006900)
Supplement: S3 Appendix — (PDF) [file pcbi.1006900.s003.pdf]

S3 Appendix Modeling noise in the target scattering data.
To assess the method's robustness towards errors in the scattering data, we conducted a structure-based LAO holo-to-apo refinement towards theoretical difference data with artificial noise. Absolute reference and target intensities were blurred according to Gaussian distributions with mean μ^q and standard deviation σ^q. For each q point, mean and standard deviation were modeled as the related clean intensity value $\mu^q = I(q)$ and its square root $\sigma^q = 50 \cdot \sqrt{I(q)}$, respectively. We calculated noisy difference data by subtracting the blurred reference intensity from the blurred target intensity (see Fig 10).
