# Supplementary material for: Rapid interpretation of small-angle X-ray scattering data
Source: PLoS Comput Biol. 2019 Mar 22;15(3):e1006900. doi: 10.1371/journal.pcbi.1006900 (PMC6447237; doi:10.1371/journal.pcbi.1006900)
Supplement: S4 Appendix — (PDF) [file pcbi.1006900.s004.pdf]

### S4 Appendix Molecular dynamics parameter file for scattering-guided SBM simulations.

```
; Sample mdp file for scattering-guided SBM simulations in GROMACS 5
;
; Run control
integrator = sd      ;leap-frog algorithm
dt         = 0.0005  ;time step (no physical SBM timescale!)
nsteps     = 4000000 ;number of steps
;
; SAXS-associated parameters
waxs-type        = Debye    ;SAXS refinement run
waxs-fc          = ???      ;coupling constant (to be inserted)
waxs-nstout      = 1        ;output frequency for SAXS intensities
waxs-nstcalc     = 10       ;frequency for updating SAXS forces
debye-alpha-mode = 0        ;constant alpha
debye-alpha-min  = 1.0
debye-alpha-max  = 1.0
;
; Output control
nstxout-compressed = 100     ;output frequency for positions (compressed)
compressed-x-grps  = Protein ;group(s) to write to compressed trajectory
nstcalcenergy      = 105     ;frequency for energy calculation
nstenergy          = 105     ;output frequency for energies (energy file)
nstlog             = 100     ;output frequency for energies (log file)
nstcomm            = 105     ;frequency for center of mass motion removal
;
; Neighbor searching
cutoff-scheme = group ;Generate pair list for atom groups.
ns-type       = grid  ;Make grid in box, check atoms in neighboring cells.
nstlist       = 15    ;frequency to update neighbor list
pbc           = xyz   ;3D periodic BC
;
; Electrostatics
coulombtype = Cut-off ;twin range cut-off
rcoulomb    = 1.5     ;Coulomb cut-off distance
;
; VdW
vdwtype = Cut-off ;twin range cut-off
rvdw    = 1.5     ;VdW cut-off distance
;
; Temperature coupling
tc-grps = Protein   ;group(s) to couple separately to temperature bath
tau-t   = 0.5       ;time constant
ref-t   = ???       ;reference temperature (not physical!; to be inserted)
;
; Pressure coupling
pcoupl = no ;no pressure coupling
;
; Velocity generation
gen-vel  = yes  ;velocity generation according to Maxwell distribution
gen-temp = ???  ;temperature for Maxwell distribution (to be inserted)
gen-seed = -1   ;Initialize velocity generator with pseudo random seed.
;
; Bonds
continuation         = no        ;Constrain start configuration.
constraint_algorithm = lincs     ;Use LINear Constraint Solver.
constraints          = all-bonds ;Convert all bonds to constraints.
lincs-iter           = 1         ;number of iterations
lincs-order          = 4         ;order of LINCS matrix expansion
```
