# Supplementary material for: Rapid interpretation of small-angle X-ray scattering data
Source: PLoS Comput Biol. 2019 Mar 22;15(3):e1006900. doi: 10.1371/journal.pcbi.1006900 (PMC6447237; doi:10.1371/journal.pcbi.1006900)
Supplement: S5 Appendix — The tutorial requires valid installations of the scattering-guided GROMACS software [20], the python package eSBMTools [30], and the molecular visualization program VMD [63]. (PDF) [file pcbi.1006900.s005.pdf]

S5 Appendix. How-to tutorial

In the following, `$` *denotes the prompt of the interactive shell used. The full prompt usually looks like* `user@machine:path$`*. Commands have to be entered in an interactive shell session. Placeholders for variables are specified in angle brackets, e.g.* `<number of cores>`*.* `>>>` *denotes the* `python` *prompt.*
For convenience, we provide all commands entered during the tutorial in a `COMMANDS` *text file (see SI attachments). To avoid unnecessary errors due to typos, just copy and paste the commands in your interactive shell session. The commands work for a unix-based operating system. Note that the* `sed` *commands may need to be modified to work properly, depending on the POSIX specifications of your operating system.*

This tutorial shows step by step how to set up and run scattering-guided SBM simulations. The aim is to provide the user with all steps principally involved in setting up a new system. As the input set and all intermediately generated output files are provided as supplementary data, prior knowledge of `GROMACS` is not absolutely necessary. The tutorial requires valid installations of `eSBMTools` [48] (available at https://sourceforge.net/projects/esbmtools/) and `GROMACS 5` including the scattering-guided MD extension [20]. `eSBMTools` is a `python 2.7` package for enhanced SBM building of proteins on the all-atom level. It interfaces with `GROMACS` and supports its standard output formats. Installation instructions can be found in the `README` and `INSTALL` files shipped with the code.
We used the scattering-guided `GROMACS` software version 5.1-dev-20150223-461b58f (patch 106). This software can be installed from a `git` repository as follows.

Install scattering-guided GROMACS software

Copy the `GROMACS` repository into the directory where the software is to be installed:

```
$ git clone https://gerrit.gromacs.org/gromacs.git
```

Fetch and check out the code:

```
$ cd gromacs
$ git fetch https://gerrit.gromacs.org/gromacs refs/changes/59/2659/106 &&
     git checkout FETCH_HEAD
```

To compile the code, create a `build` and an `install` directory:

```
$ cd ..
$ mkdir build
$ mkdir install
```

Run `cmake` and `make`. The exact options depend on both your computer and preferences. Note that the scattering-guided code does not support parallel MPI communication. Like normal `GROMACS`, the installation requires a Fast-Fourier-Transform library such as FFTW (see installation instructions for `GROMACS 5.1` at http://manual.gromacs.org/documentation/5.1/install-guide/index.html). To automatically download and build FFTW from source, set `DGMX_BUILD_OWN_FFTW` to `ON`. The option `-j` allows the software to be built in parallel, using the number of cores specified:

```
$ cd build
$ cmake ../gromacs -DGMX_MPI=OFF -DGMX_THREAD_MPI=OFF -DGMX_OPENMP=ON -
    DCMAKE_INSTALL_PREFIX=../install -DGMX_BUILD_OWN_FFTW=ON
$ make install -j <number of cores>
```

If `cmake` produces an error related to `libxml2`, install the `libxml2-dev` package and try again. To use the freshly installed `GROMACS` version, load the file `GMXRC` from the `install` directory into your current shell session:

```
$ source ../install/bin/GMXRC
```

Performing a simulation in GROMACS requires three different files: a molecular dynamics parameter file .mdp, a topology file .top, and a coordinate file .gro. The .mdp file contains all parameters defining the simulation settings, e.g. time step, number of steps, temperature, etc. (see S4 Appendix). The .top file sets all specifics of the considered Hamiltonian, i.e. all interactions defining the SBM. The .gro file gives the system's initial atomic coordinates according to the provided PDB structure and defines the edge lengths of the simulation box in the last line.

Set up a scattering-guided SBM simulation

To run a scattering-guided SBM simulation, consider a two-state protein system with two structurally different conformations initial.pdb and target.pdb. In the SI attachments, we provide all input and intermediately generated files for the elongated-to-bent transition in the small VHP-based test system.

Prepare the structure-based model

As a first step, set up the SBM from the initial elongated structure. Switch to the working directory containing initial.pdb, which is tut/ in the provided material. Using eSBMTools in python 2.7, build an SBM of this structure via:

```
>>> from eSBMTools import GoModel as go
>>> go.createGoModel('./', 'initial', moleculeType='AA', xmlFileName='
    aminoAcids.xml', groFileName='initial.gro', topFileName='initial.top')
```

This function takes the input structure file initial.pdb and converts it into a fully functional structure-based topology and coordinate file, initial.gro and initial.top, respectively. Possible warnings about dihedrals, bonds, or angles not found in the structure can be ignored. Also create a coordinate file for the target structure target.pdb, as we will need the file in the analysis later on:

```
>>> go.createGroFile('./', go.parsePdbFile('./','target'), 'target',
    groFileName='target.gro')
```

To make sure the SBM has been set up properly, you can inspect the coordinate file for example in VMD (download via http://www.ks.uiuc.edu/Research/vmd/) [63]:

```
$ vmd initial.gro
```

When generating the scattering topology in the next step, a special atom type MW is used to represent virtual scattering sites in the structure. As it is not contained in the SBM by default, we have to manually include it. To do so, add the following line

```
MW       0.000     0.000 A     0.000  0.000
```

to the [atomtypes] section in the topology file initial.top, e.g. by:

```
$ sed -i -e '/moleculetype/i\ MW       0.000     0.000 A     0.000  0.000'
     -n -e '/MW/{x;d;};1h;1!{x;p;};${x;p;}' initial.top
```

You can check the modifications to initial.top with your favorite text editor.

Prepare the scattering input

Build the scattering topology required for calculation of SAXS curves from biomolecular structures during the simulation with gmx genrestr. By specifying the help option -h, you can get more information on all GROMACS gmx tools. Use amino-acid based scattering factors with displaced-solvent correction:

```
$ gmx genrestr -matrix residue -d $GMXDATA/top/
    sfactor_amino_acid_ds_Fourier.xml -o saxs.itp -f initial.gro -oc
    saxsInitial.gro
```

This will produce a topology include file `saxs.itp` and an extended coordinate file `saxsInitial.gro`, specifying the virtual scattering sites in the structure. Notifications on unknown units can be ignored. Include the `.itp` file containing the scattering topology into the SBM topology file `initial.top`. To do so, add

```
#include "saxs.itp"
```

directly after the `[ atoms ]` section and name the resulting file `saxsInitial.top`, e.g. by:

```
$ sed -e '/\[ pairs \]/i\ #include "saxs.itp"' -n -e '/saxs/{x;d;};1h;1!{x
    ;p;};${x;p;}' initial.top > saxsInitial.top
```

You can inspect the generated file `saxsInitial.top` in your favorite text editor. Preprocess the system with `gmx grompp` to obtain the run input file `run.tpr`:

```
$ gmx grompp -f run.mdp -c saxsInitial.gro -p saxsInitial.top -o run.tpr
```

The `.tpr` file extension stands for portable binary run input file. Such files combine the simulation's initial structure, the molecular topology, and all simulation parameters. Generate the reference scattering from the initial structure using `gmx waxsdebye`:

```
$ gmx waxsdebye -s run.tpr -waxs_ref saxs_zeros.dat -sfac $GMXDATA/top/
    sfactor_amino_acid_ds_Fourier.xml -waxs_out saxs_initial
```

Remove lines starting with @ and & from `saxs_initial.xvg`, delete the third column, and rename the file `saxs_initial.dat`, e.g. by:

```
sed -e '/@\|&/d' -e 's/^ *//' -e 's/  / /g' saxs_initial.xvg | cut -d" " -
    f1,2 > saxs_initial.dat
```

Check the reference scattering data file `saxs_initial.dat` with your favorite text editor.

Run the simulation

Run the SBM refinement with `gmx mdrun`. On an average computer, the simulation will run approximately ten minutes. Important parameters are temperature `ref-t` and the coupling to the scattering data `waxs-fc`. Use `saxs_initial.dat` and `saxs_diff.dat` as reference and target scattering data in the simulation, respectively:

```
$ gmx mdrun -v -deffnm run -waxs_ref saxs_initial.dat -waxs_diff saxs_diff
    .dat -sfac $GMXDATA/top/sfactor_amino_acid_ds_Fourier.xml -waxs_out
    saxs_out -waxs_alpha saxs_alpha
```

This will produce a compressed trajectory `run.xtc`, the simulation's final frame `run.gro`, scattering curves `saxs_out.xvg` calculated during the refinement, an energy file `run.edr`, and a log file `run.log`. The `.trr` trajectory as well as `.cpt` checkpoint files are only important for doing simulation restarts and extensions. As α is kept constant during the refinement, the output file `saxs_alpha.xvg` is of no further interest.

Analyze the results

To view the resulting trajectory as a cartoon in `VMD`, we have to remove the virtual scattering sites and choose `Protein`:

```
echo 1 | gmx trjconv -s initial.gro -f run.xtc -o run_processed.xtc
```

Compare the simulation to the original initial (and target) structure. Do the RMSD analysis on C_α level and extract the bias energy term from the `GROMACS` energy output file `run.edr`:

```
echo 3 3 | gmx rms -s initial.gro -f run_processed.xtc -o rmsd_initial
echo 3 3 | gmx rms -s target.gro -f run_processed.xtc -o rmsd_target
echo 10 | gmx energy -f run.edr -o V_XS
```

View the results `.xvg` in e.g. `xmgrace`:

```
xmgrace rmsd_initial.xvg rmsd_target.xvg
xmgrace V_XS.xvg
```
