# Supplementary figures and images for: Rapid interpretation of small-angle X-ray scattering data
Source: PLoS Comput Biol. 2019 Mar 22;15(3):e1006900. doi: 10.1371/journal.pcbi.1006900 (PMC6447237; doi:10.1371/journal.pcbi.1006900)

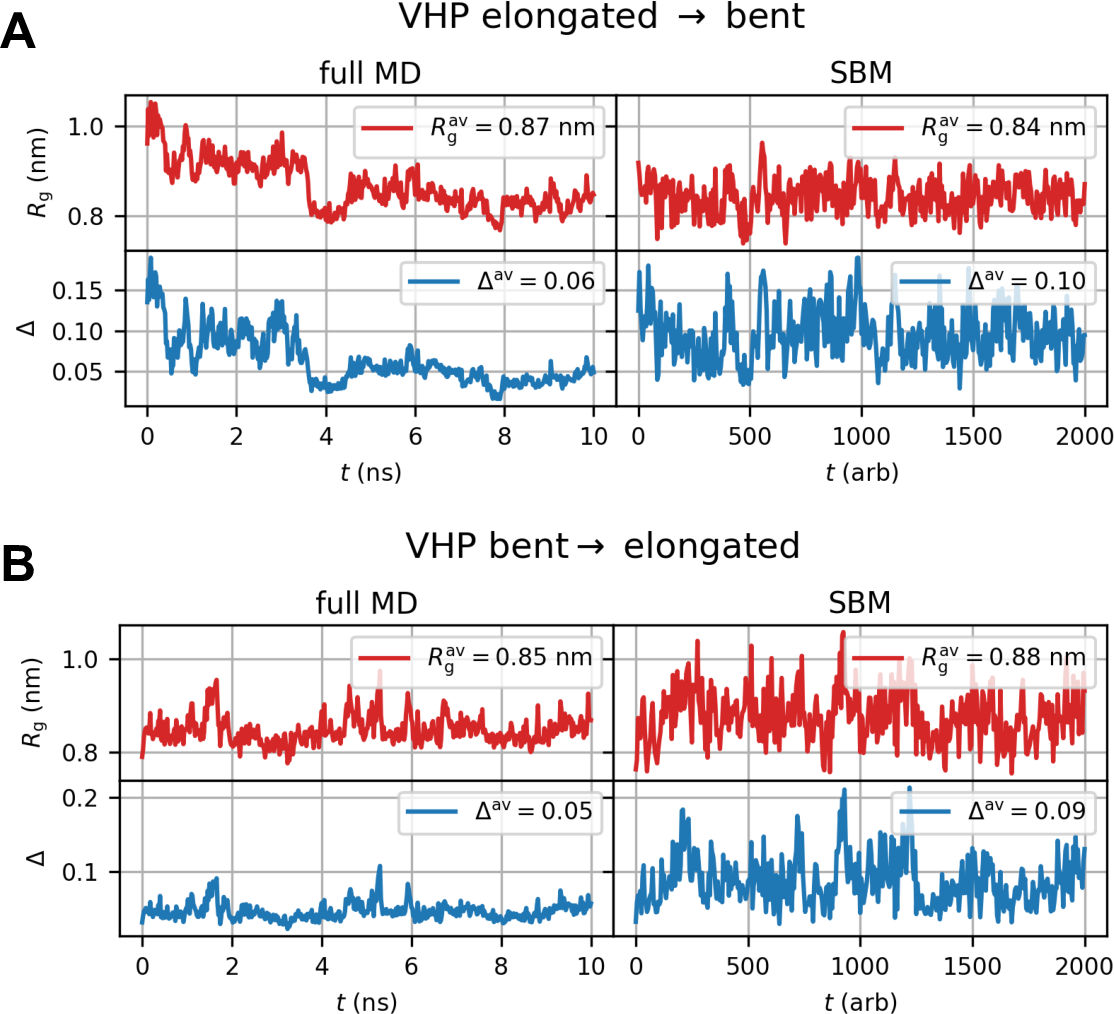

Supplement: S1 Fig — Shape parameters for VHP elongated-to-bent and bent-to-elongated transition (A and B, respectively). Radius of gyration (red) and asphericity (blue) versus simulated time are shown at the top and bottom of each panel. Results at the left and right of each panel belong to scattering-guided full-MD and SBM simulations, respectively. (TIF) [file pcbi.1006900.s006.tif]

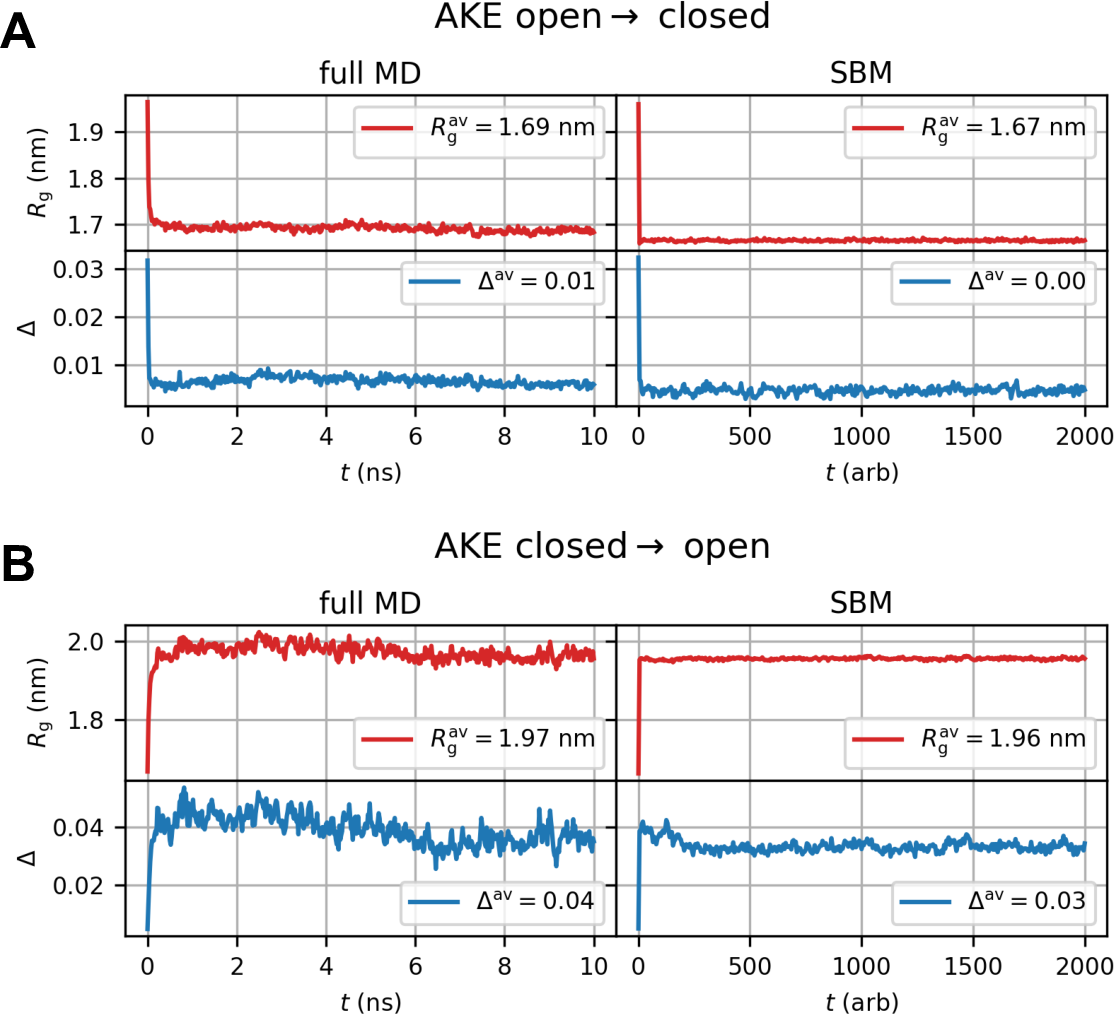

Supplement: S2 Fig — Shape parameters for AKE open-to-closed and closed-to-open transition (A and B, respectively). Radius of gyration (red) and asphericity (blue) versus simulated time are shown at the top and bottom of each panel. Results at the left and right of each panel belong to scattering-guided full-MD and SBM simulations, respectively. (TIF) [file pcbi.1006900.s007.tif]

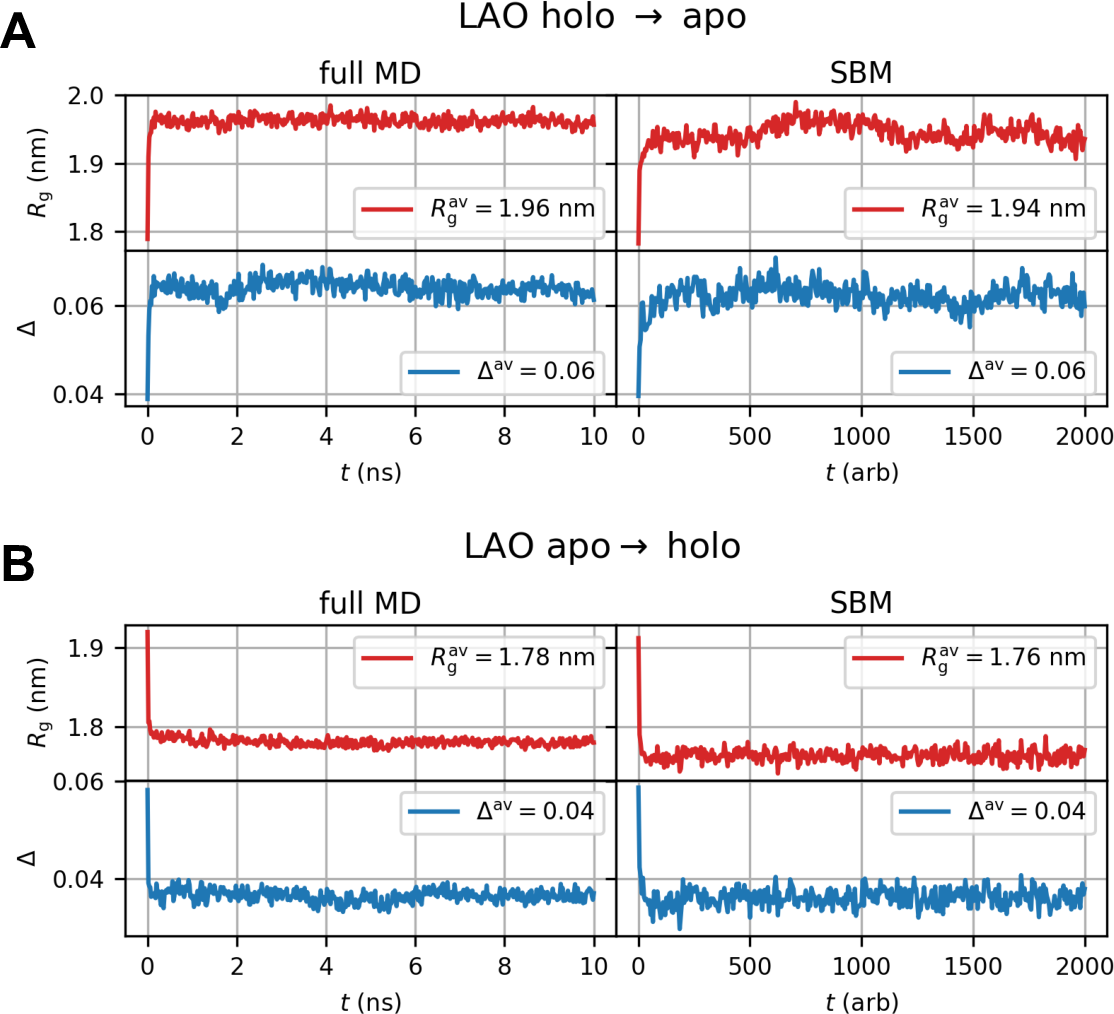

Supplement: S3 Fig — Shape parameters for LAO holo-to-apo and apo-to-holo transition (A and B, respectively). Radius of gyration (red) and asphericity (blue) versus simulated time are shown at the top and bottom of each panel. Results at the left and right of each panel belong to scattering-guided full-MD and SBM simulations, respectively. (TIF) [file pcbi.1006900.s008.tif]

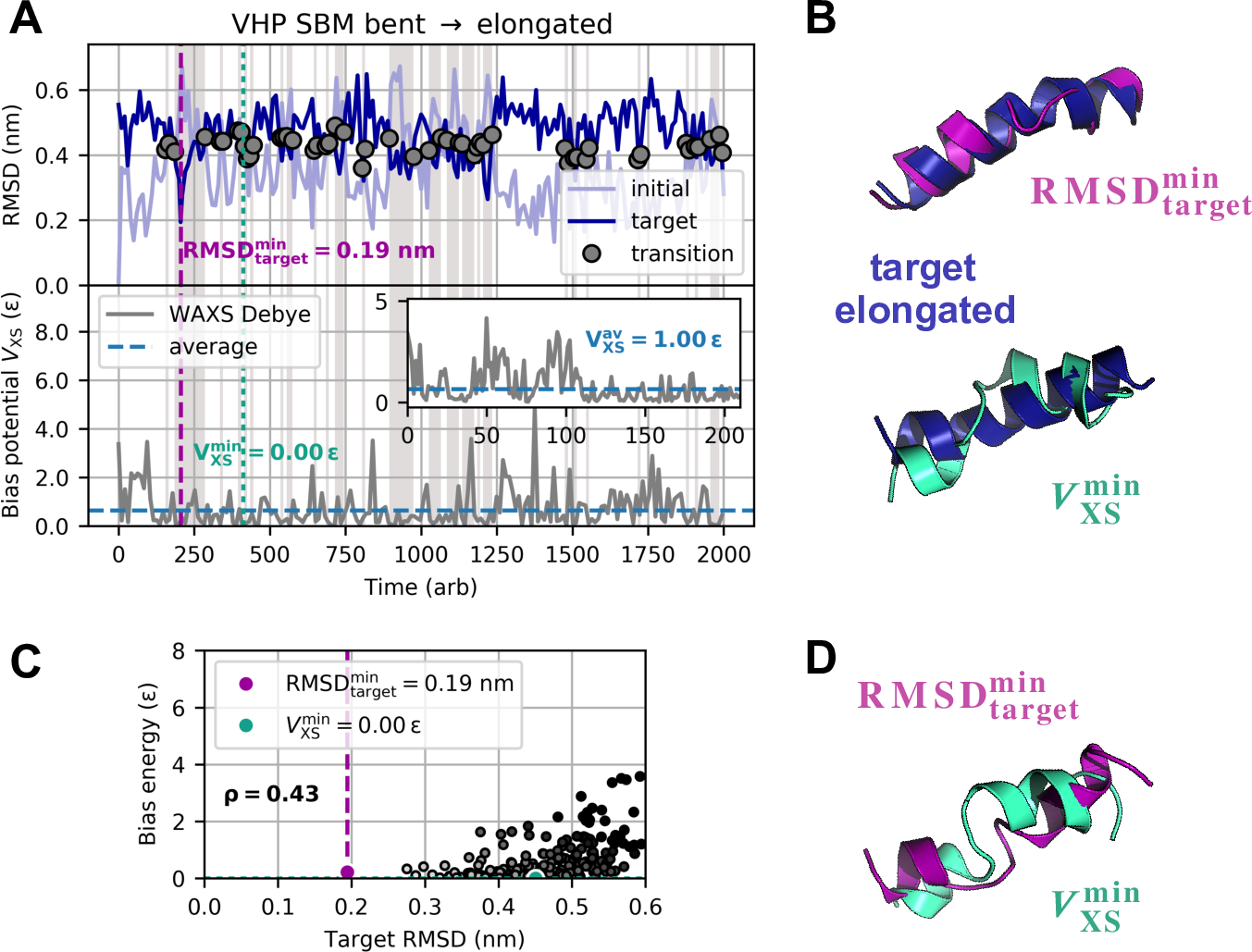

Supplement: S4 Fig — Results are shown for parameters (T, kχ) = (90, 5 ⋅ 10−8 ε). (A) Initial and target RMSD (top) and bias energy (bottom) versus simulated time. (B) Best structures as measured by target RMSD and bias energy. RMSDtargetmin structure (purple) and VXSmin structure (turquoise) feature target RMSDs of 0.19 nm and 0.37 nm, respectively. (C) Bias potential versus target RMSD. (D) RMSDtargetmin (purple) and VXSmin (turquoise) structure exhibit an RMSD of 0.31 nm with respect to each other. (TIF) [file pcbi.1006900.s009.tif]

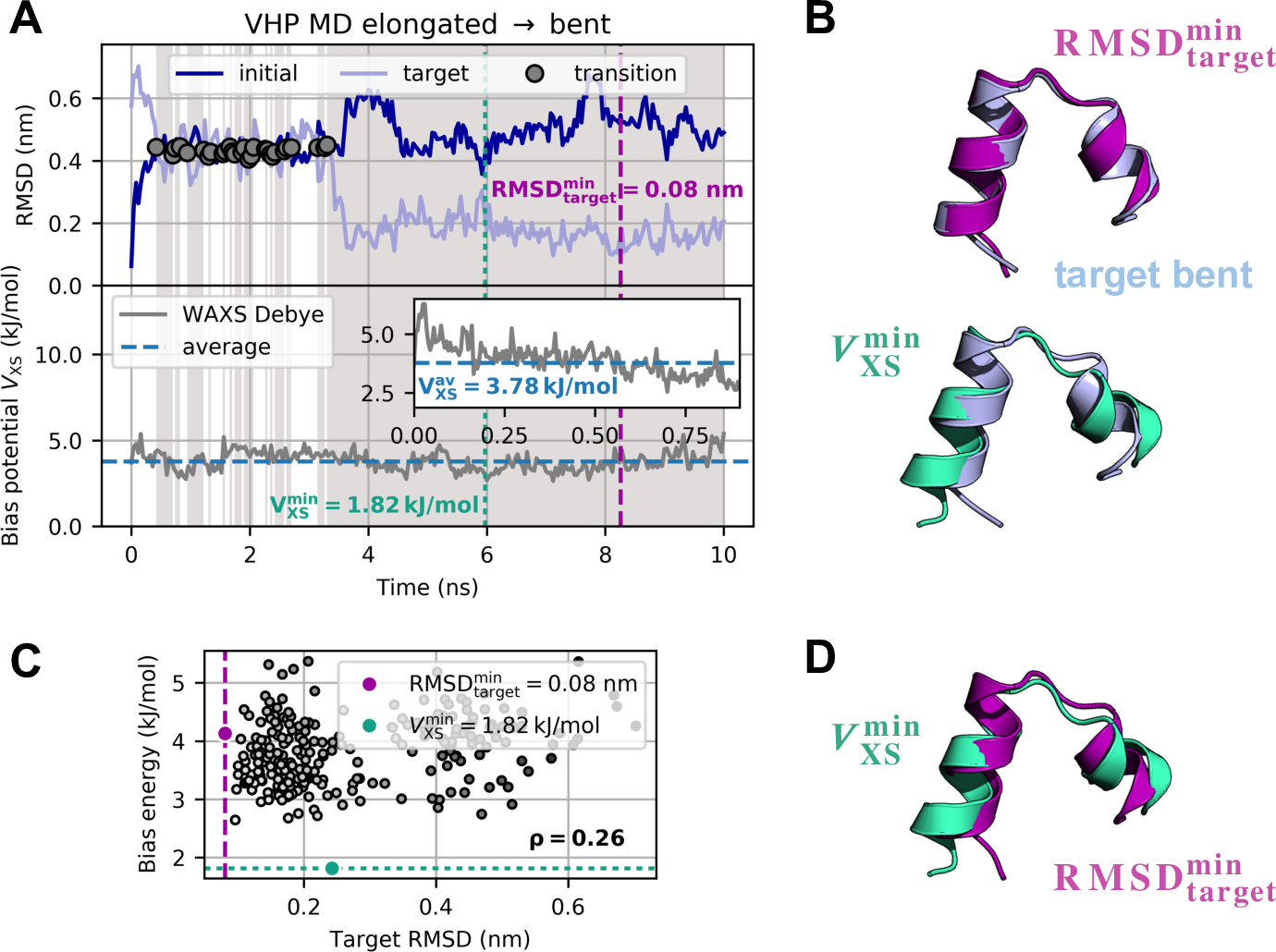

Supplement: S5 Fig — Results are shown for parameters (T, kχ) = (330 K, 5 ⋅ 10−9 kJ/mol). (A) Initial and target RMSD (top) and bias energy (bottom) versus simulated time. (B) Best structures as measured by target RMSD and bias energy. RMSDtargetmin structure (purple) and VXSmin structure (turquoise) feature target RMSDs of 0.08 nm and 0.24 nm, respectively. (C) Bias potential versus target RMSD. (D) RMSDtargetmin (purple) and VXSmin (turquoise) structure exhibit an RMSD of 0.21 nm with respect to each other. (TIF) [file pcbi.1006900.s010.tif]

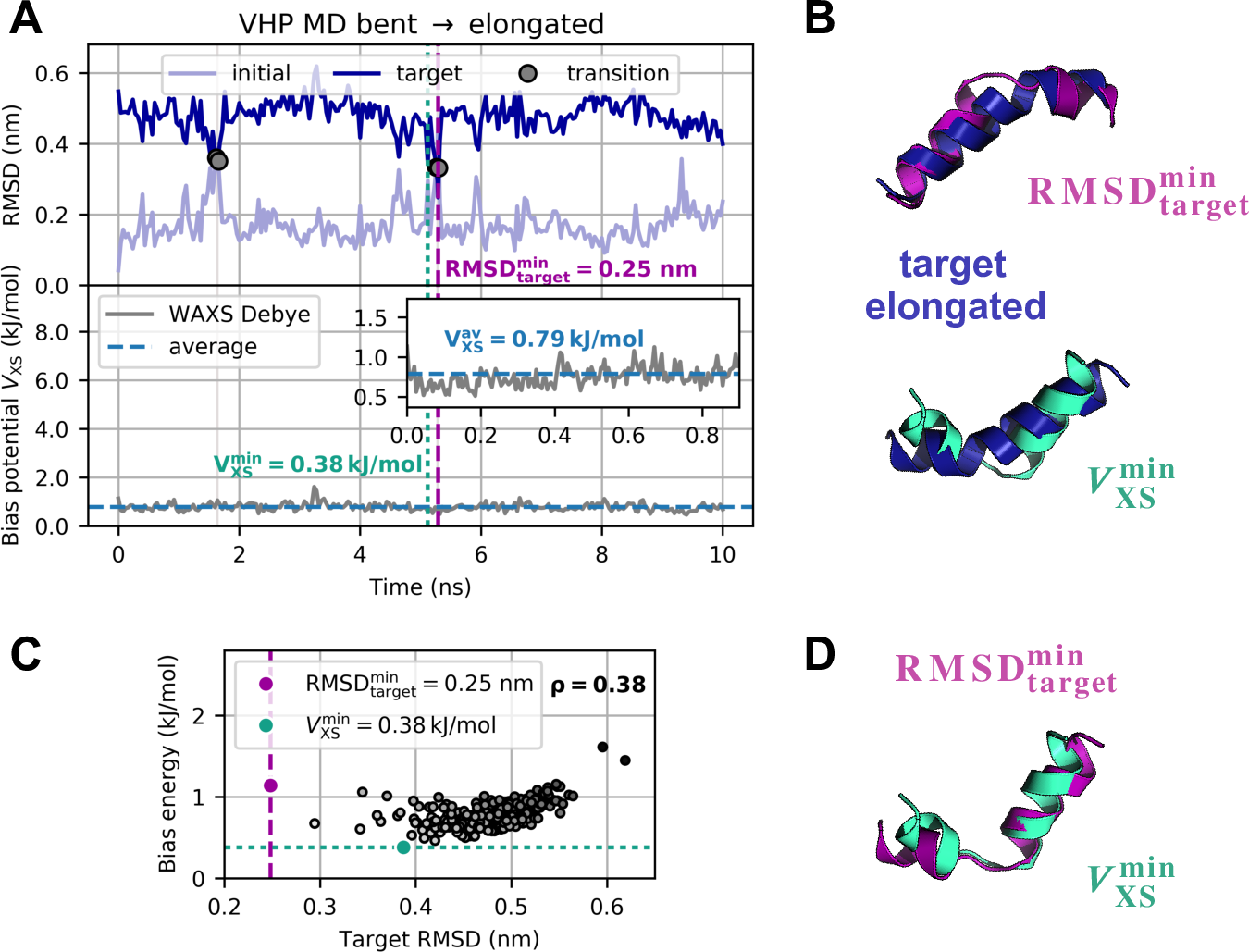

Supplement: S6 Fig — Results are shown for parameters (T, kχ) = (330 K, 5 ⋅ 10−9 kJ/mol). (A) Initial and target RMSD (top) and bias energy (bottom) versus simulated time. Apparently, the simulation could only selectively sample conformations near the target structure. (B) Best structures as measured by target RMSD and bias energy. RMSDtargetmin structure (purple) and VXSmin structure (turquoise) feature target RMSDs of 0.25 nm and 0.39 nm, respectively. (C) Bias potential versus target RMSD. (D) RMSDtargetmin (purple) and VXSmin (turquoise) structure exhibit an RMSD of 0.28 nm with respect to each other. (TIF) [file pcbi.1006900.s011.tif]

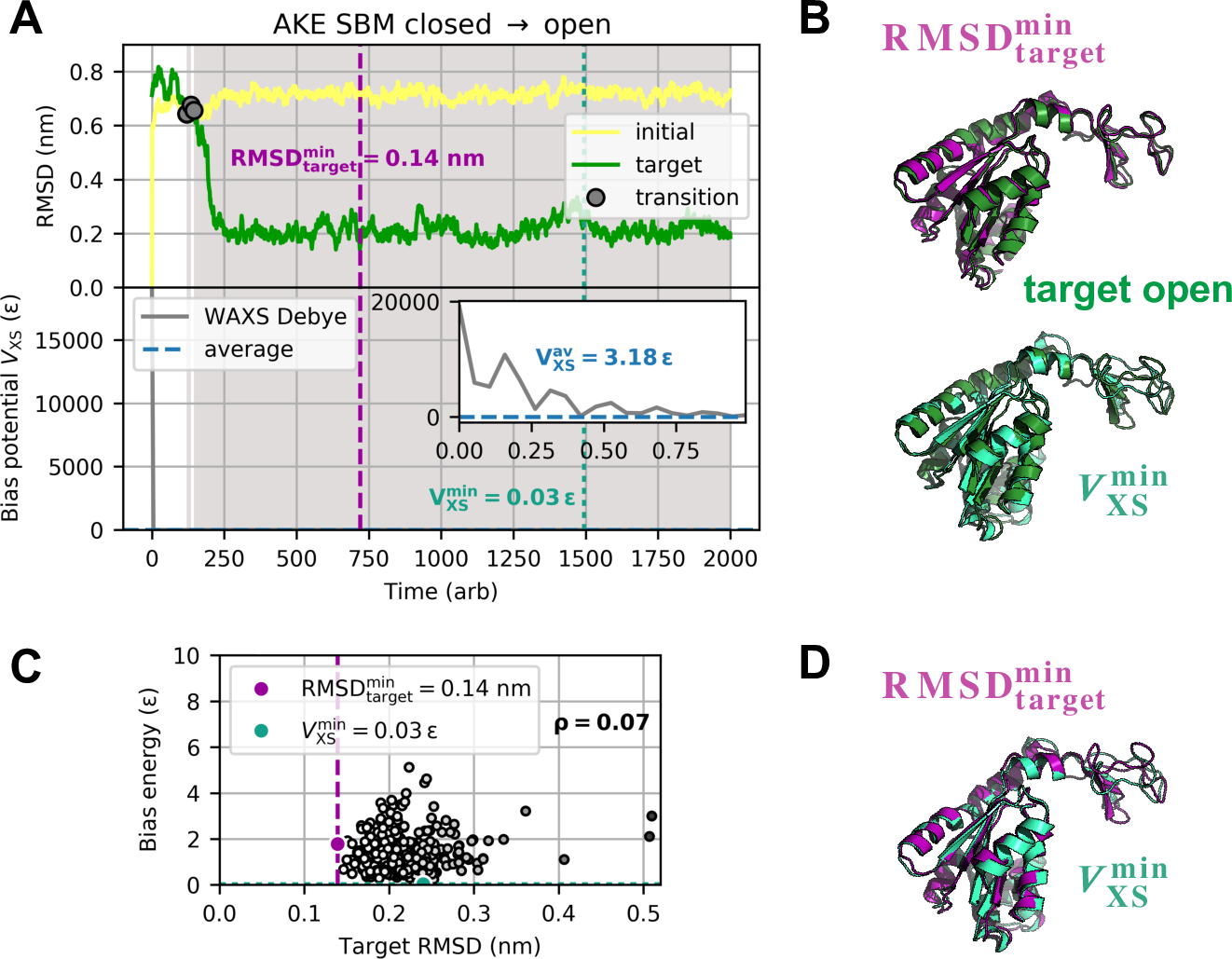

Supplement: S7 Fig — Results are shown for parameters (T, kχ) = (50, 8 ⋅ 10−9 ε). (A) Initial and target RMSD (top) and bias energy (bottom) versus simulated time. (B) Best structures as measured by target RMSD and bias energy. RMSDtargetmin structure (purple) and VXSmin structure (turquoise) have target RMSDs of 0.14 nm and 0.24 nm, respectively. (C) Bias potential versus target RMSD. (D) RMSDtargetmin (purple) and VXSmin (turquoise) structure exhibit an RMSD of 0.22 nm with respect to each other. (TIF) [file pcbi.1006900.s012.tif]

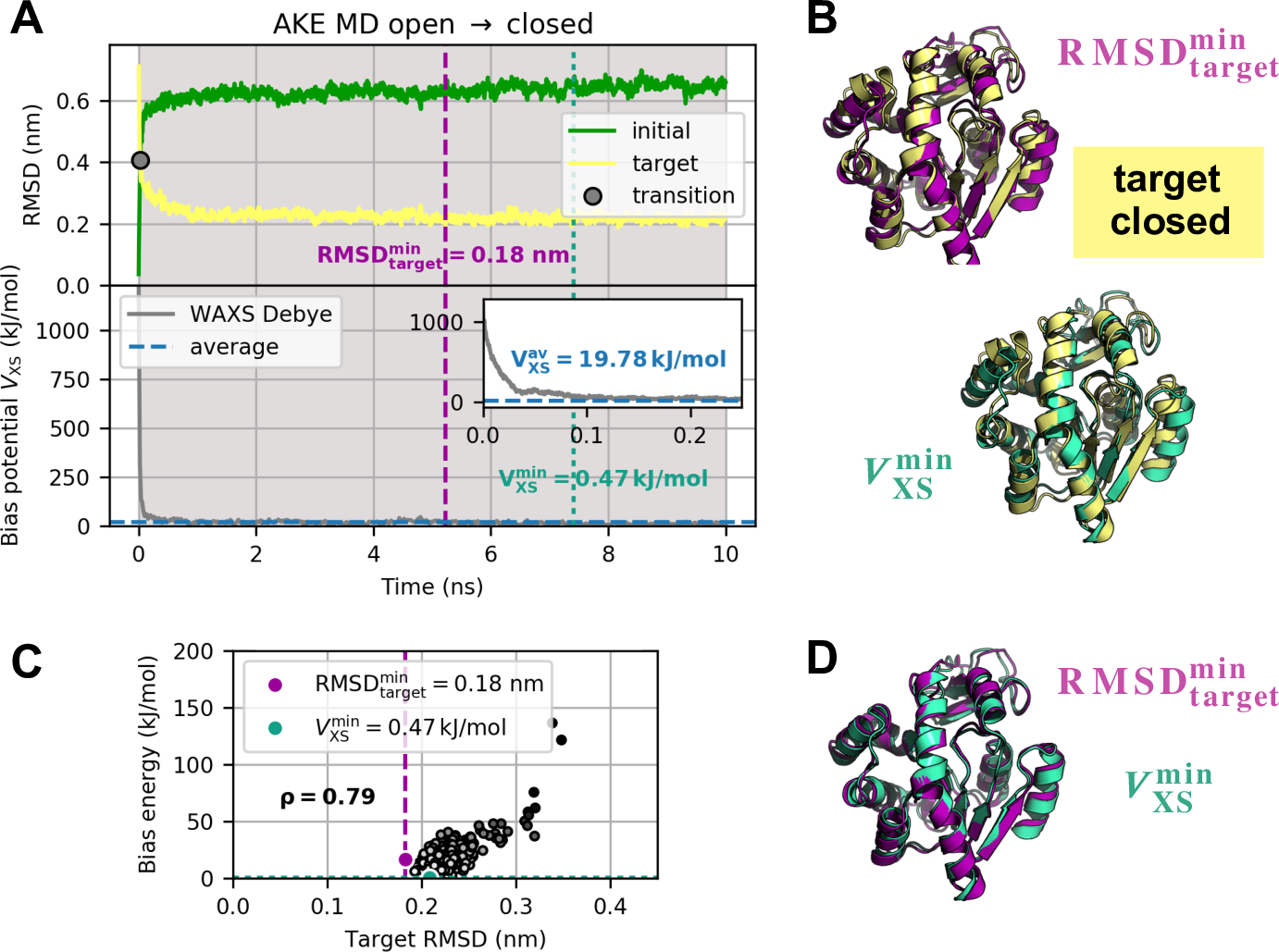

Supplement: S8 Fig — Results are shown for parameters (T, kχ) = (300 K, 5 ⋅ 10−10 kJ/mol). (A) Initial and target RMSD (top) and bias energy (bottom) versus simulated time. (B) Best structures as measured by target RMSD and bias energy. RMSDtargetmin structure (purple) and VXSmin structure (turquoise) have target RMSDs of 0.18 nm and 0.21 nm, respectively. (C) Bias potential versus target RMSD. (D) RMSDtargetmin (purple) and VXSmin (turquoise) structure exhibit an RMSD of 0.11 nm with respect to each other. (TIF) [file pcbi.1006900.s013.tif]

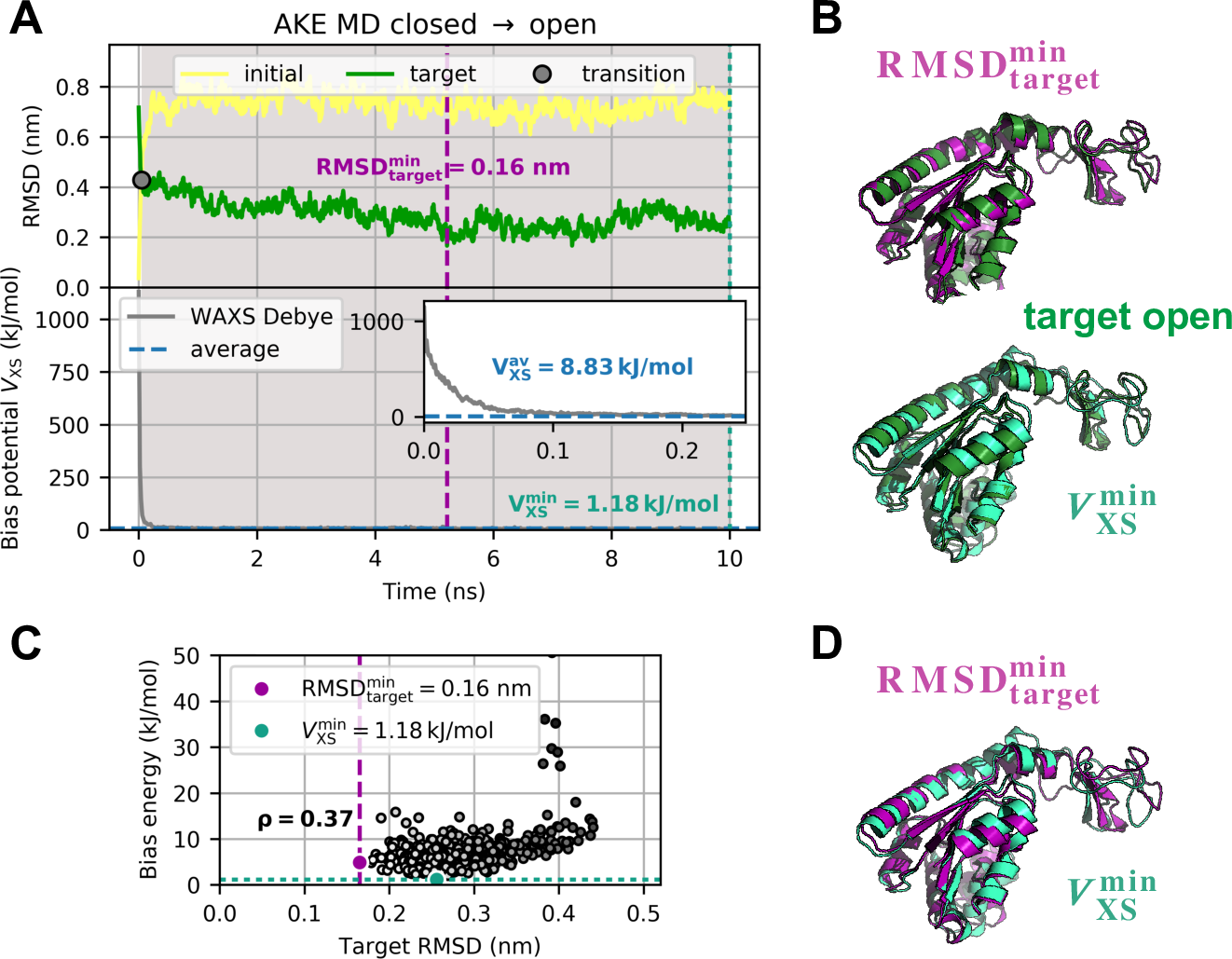

Supplement: S9 Fig — Results are shown for parameters (T, kχ) = (300 K, 5 ⋅ 10−10 kJ/mol). (A) Initial and target RMSD (top) and bias energy (bottom) versus simulated time. (B) Best structures as measured by target RMSD and bias energy. RMSDtargetmin structure (purple) and VXSmin structure (turquoise) have target RMSDs of 0.16 nm and 0.26 nm, respectively. (C) Bias potential versus target RMSD. (D) RMSDtargetmin (purple) and VXSmin (turquoise) structure exhibit an RMSD of 0.29 nm with respect to each other. (TIF) [file pcbi.1006900.s014.tif]

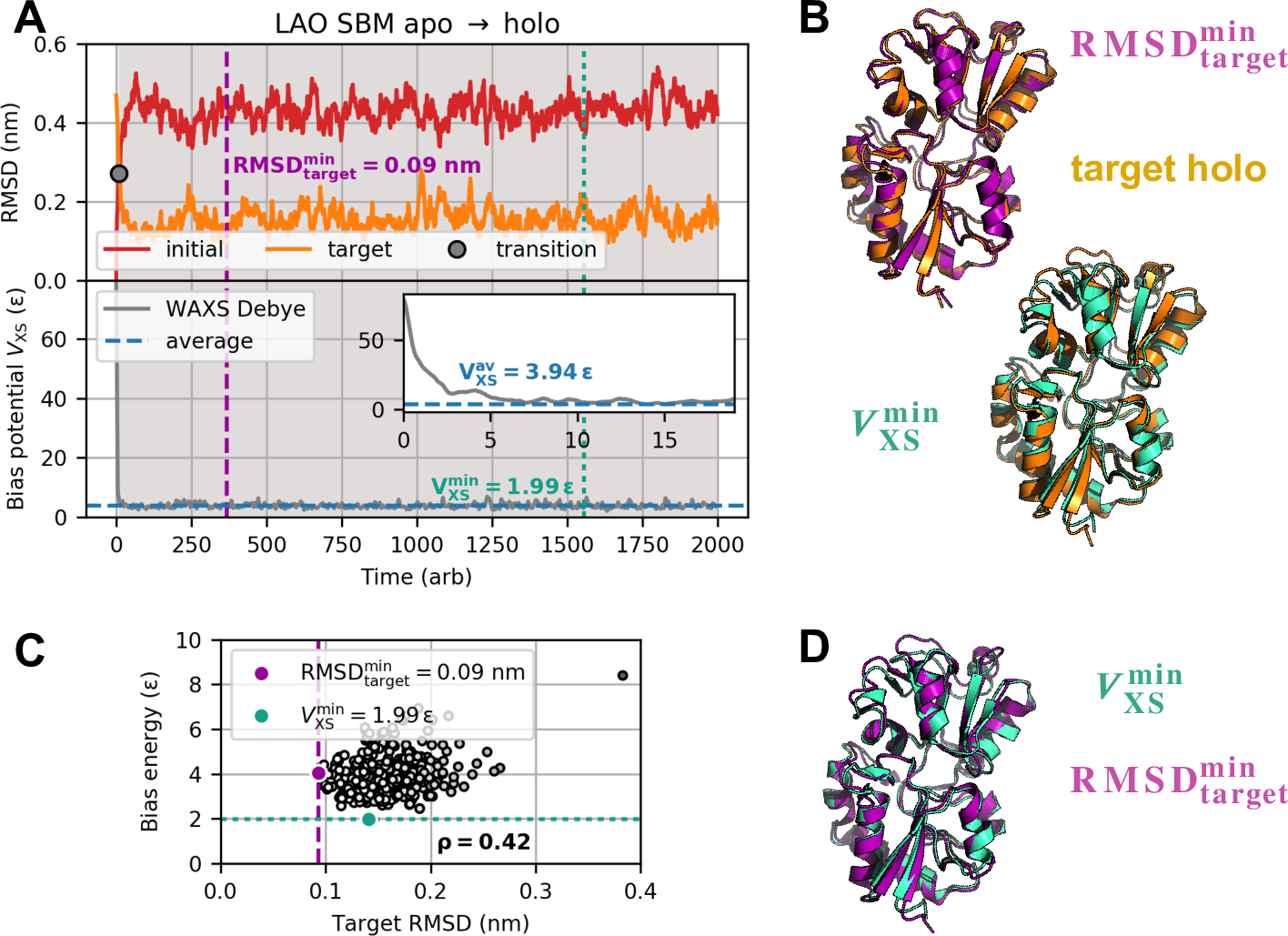

Supplement: S10 Fig — Results are shown for parameters (T, kχ) = (50, 9 ⋅ 10−11 ε). (A) Initial and target RMSD (top) and bias energy (bottom) versus simulated time. (B) Best structures as measured by target RMSD and bias energy. RMSDtargetmin structure (purple) and VXSmin structure (turquoise) have target RMSDs of 0.09 nm and 0.14 nm, respectively. (C) Bias potential versus target RMSD. (D) RMSDtargetmin (purple) and VXSmin (turquoise) structure exhibit an RMSD of 0.13 nm with respect to each other. (TIF) [file pcbi.1006900.s015.tif]

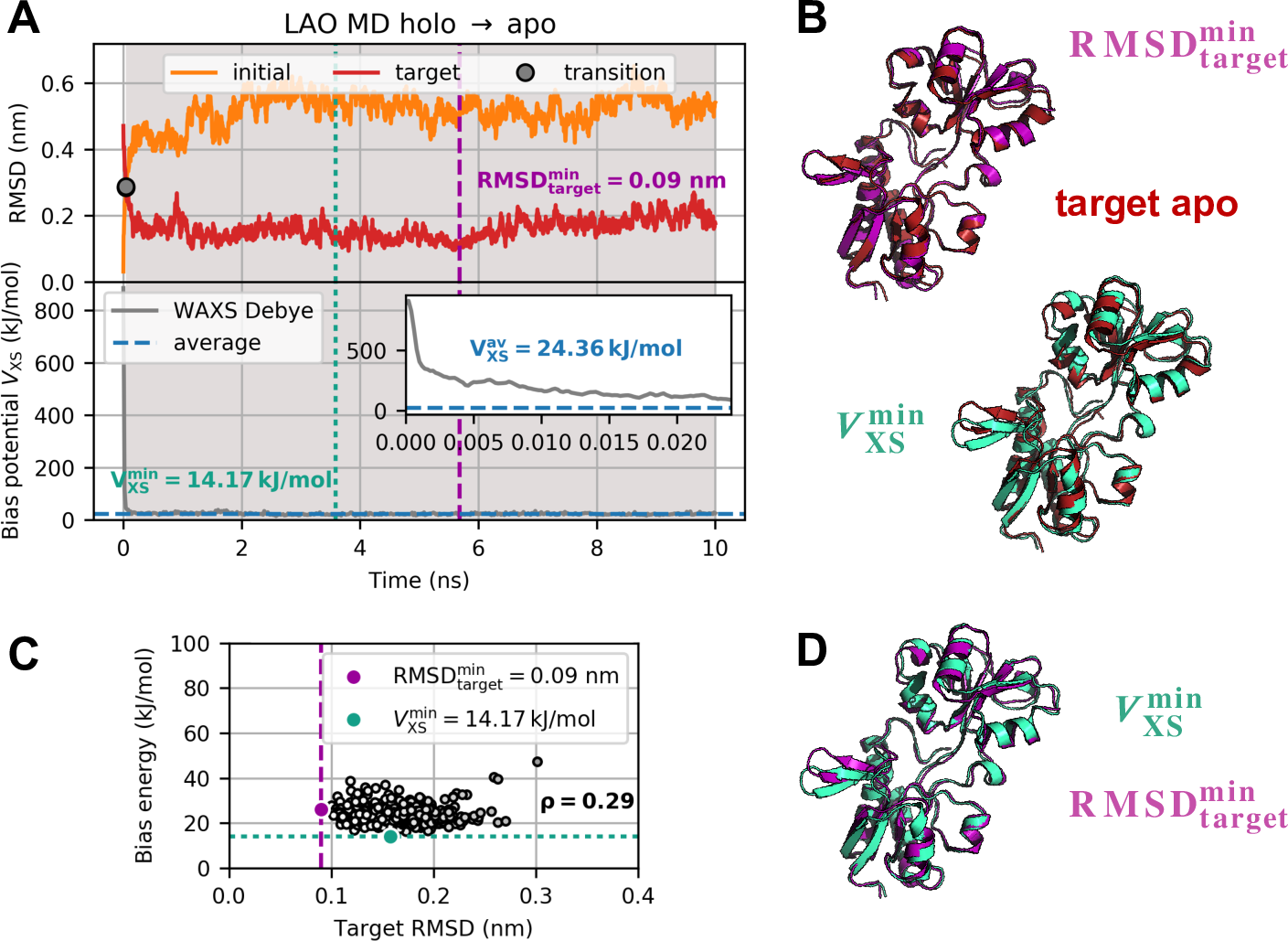

Supplement: S11 Fig — Results are shown for parameters (T, kχ) = (300 K, 1 ⋅ 10−9 kJ/mol). (A) Initial and target RMSD (top) and bias energy (bottom) versus simulated time. (B) Best structures as measured by target RMSD and bias energy. RMSDtargetmin structure (purple) and VXSmin structure (turquoise) have target RMSDs of 0.09 nm and 0.16 nm, respectively. (C) Bias potential versus target RMSD. (D) RMSDtargetmin (purple) and VXSmin (turquoise) structure exhibit an RMSD of 0.14 nm with respect to each other. (TIF) [file pcbi.1006900.s016.tif]

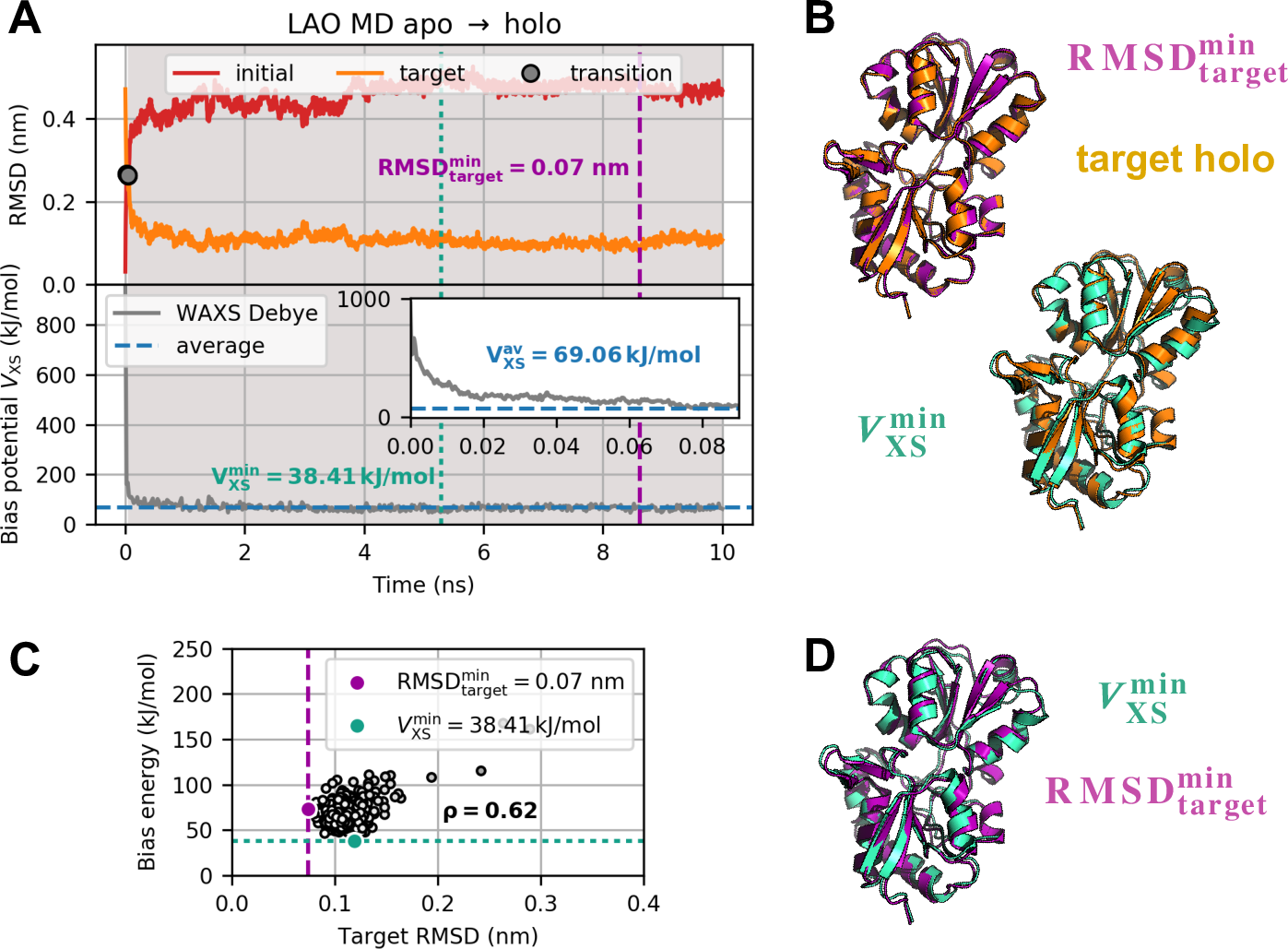

Supplement: S12 Fig — Results are shown for parameters (T, kχ) = (300 K, 1 ⋅ 10−9 kJ/mol). (A) Initial and target RMSD (top) and bias energy (bottom) versus simulated time. (B) Best structures as measured by target RMSD and bias energy. RMSDtargetmin structure (purple) and VXSmin structure (turquoise) have target RMSDs of 0.07 nm and 0.12 nm, respectively. (C) Bias potential versus target RMSD. (D) RMSDtargetmin (purple) and VXSmin (turquoise) structure exhibit an RMSD of 0.11 nm with respect to each other. (TIF) [file pcbi.1006900.s017.tif]

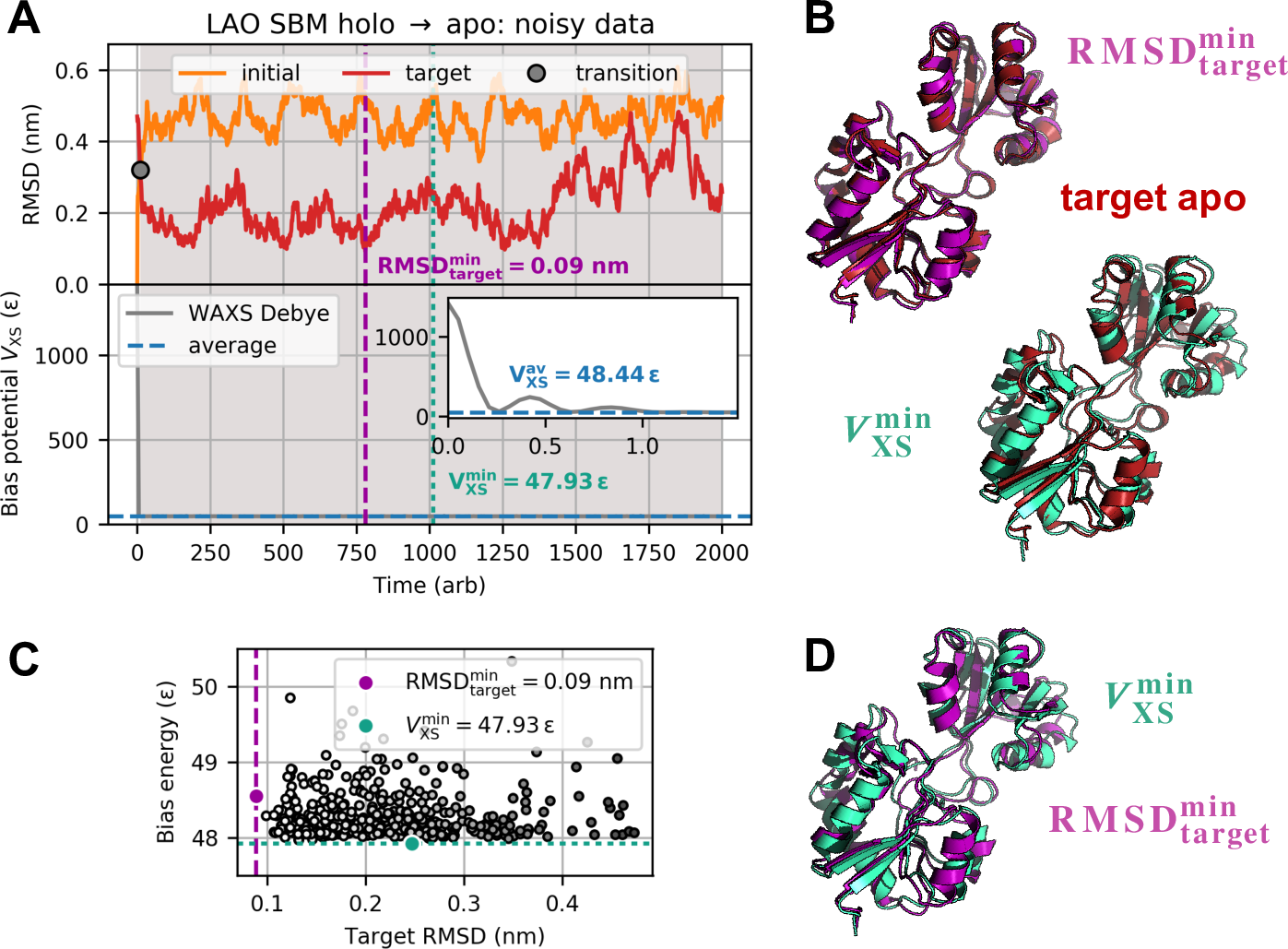

Supplement: S13 Fig — (A) Initial and target RMSD (top) and bias energy (bottom) versus simulated time. (B) Best structures as measured by target RMSD and bias energy. RMSDtargetmin structure (purple) and VXSmin structure (turquoise) have target RMSDs of 0.09 nm and 0.25 nm, respectively. (C) Bias potential versus target RMSD. (D) RMSDtargetmin (purple) and VXSmin (turquoise) structure exhibit an RMSD of 0.25 nm with respect to each other. (TIF) [file pcbi.1006900.s018.tif]
